# Integrative taxonomic approach to the cryptic diversity of *Diplostomum* spp. in lymnaeid snails from Europe with a focus on the ‘*Diplostomum mergi*’ species complex

**DOI:** 10.1186/s13071-015-0904-4

**Published:** 2015-06-03

**Authors:** Christian Selbach, Miroslava Soldánová, Simona Georgieva, Aneta Kostadinova, Bernd Sures

**Affiliations:** Department of Aquatic Ecology and Centre for Water and Environmental Research (ZWU), University of Duisburg-Essen, Universitätsstraße 5, D-45141 Essen, Germany; Institute of Parasitology, Biology Centre of the Czech Academy of Sciences, Branišovská 31, 370 05 České Budějovice, Czech Republic; Department of Zoology, University of Johannesburg, Auckland Park 2006, PO Box 524, Johannesburg, South Africa

**Keywords:** ‘*Diplostomum mergi*’ species complex, *Diplostomum parviventosum*, *Diplostomum pseudospathaceum*, *Diplostomum spathaceum*, *Radix auricularia*, *Lymnaea stagnalis*, *Stagnicola palustris*, Cercariae, *cox*1, ITS, Europe

## Abstract

**Background:**

Recent molecular studies have discovered substantial unrecognised diversity within the genus *Diplostomum* in fish populations in Europe and North America including three species complexes. However, data from the first intermediate host populations are virtually lacking. This study addresses the application of an integrative taxonomic approach to the cryptic species diversity of *Diplostomum* spp. in natural lymnaeid snail populations in Europe with a focus on the ‘*D. mergi*’ species complex.

**Methods:**

Totals of 1,909 *Radix auricularia*, 349 *Radix peregra*, 668 *Stagnicola palustris* and 245 *Lymnaea stagnalis* were sampled at five reservoirs of the Ruhr river system in Germany and screened for infections with *Diplostomum* spp. Cercariae were examined and identified alive, fixed and under scanning electron microscopy. Sequences from the barcode region of the cytochrome *c* oxidase subunit 1 (*cox*1) mitochondrial gene and from the internal transcribed spacer cluster (ITS1-5.8S-ITS2) of the rRNA gene were amplified for 51 and 13 isolates, respectively.

**Results:**

Detailed morphological and molecular analyses provided evidence for three named species (*Diplostomum spathaceum*, *D. pseudospathaceum* and *D. parviventosum*), and a further four species-level lineages (‘*D. mergi* Lineages 2–4’ and ‘*Diplostomum* sp. Clade Q’ in the lymnaeid snail populations from the Ruhr river basin. The paper provides the first descriptions of molecularly identified cercariae of *D. spathaceum* and of the cercariae of *D. parviventosum*, three lineages of the ‘*D. mergi*’ species complex and of ‘*Diplostomum* sp. Clade Q’.

**Conclusion:**

The integration of molecular and morphological evidence for *Diplostomum* spp. achieved in this study will serve as a baseline for species identification of these important parasites of snail and fish populations and thus advance further studies on the distribution of *Diplostomum* spp. in Europe.

**Electronic supplementary material:**

The online version of this article (doi:10.1186/s13071-015-0904-4) contains supplementary material, which is available to authorized users.

## Background

The incorporation of molecular data has brought a major advancement in species taxonomy, due to the possibility to distinguish cryptic species and re-evaluate existing morphological identification criteria. Especially for trematode species with complex life-cycles, where sampling often provides only one stage of a parasite’s life-cycle at a time (*e.g.* cercariae or metacercariae only), molecular analyses provide an effective means of species identification and inference of complete life-cycles by matching data from the different life-cycle stages [1, 2]. However, although larval trematodes in snails are potentially useful indicators of environmental conditions [3], they are difficult to identify and the taxonomic expertise is limited to few individuals [4]. This highlights the importance of providing accurate and accessible information on their identification.

*Diplostomum* von Nordmann, 1832 is a major and important taxonomic group of widely distributed freshwater trematode parasites that utilise lymnaeid snails, fish and fish-eating birds to complete their life-cycles. Recent molecular studies have discovered higher than previously recognised diversity of *Diplostomum* spp. at small geographical scales. A total of 12 species was found in fishes in northern Canada based on molecular evidence: three named species, *Diplostomum indistinctum* (Guberlet, 1923), *Diplostomum huronense* (La Rue, 1927) and *Diplostomum baeri* Dubois, 1937, and a further nine unidentified species-level lineages of *Diplostomum* [5]. The first studies addressing integration of morphological assessment and molecular prospecting for species diversity of the genus in Europe resulted in molecular elucidation of the life-cycles of *Diplostomum spathaceum* (Rudolphi, 1819) (type-species) and *D. pseudospathaceum* Niewiadomska, 1984 and provided evidence for a substantial unrecognised genetic and morphological diversity, *i.e.* 15 species-level lineages including three complexes of genetically distinct lineages [6–9].

Six of these originate from the snail and fish populations studied in the River Ruhr drainage in Germany. Of particular importance is the finding of a number of isolates comprising three slightly divergent lineages within the ‘*D. mergi*’ species complex [6]. However, most of these isolates, *i.e.* ‘*D. mergi* Lineage 3’, originate from fish and due to the low sampling effort only few isolates from their lymnaeid snail hosts are available: a single isolate for ‘*D. mergi* Lineage 1’ and three isolates for ‘*D. mergi* Lineage 2’, all from *Radix auricularia* (L.).

The application of barcoding approach to species diversity of *Diplostomum* in Europe depends on the availability of sequence databases based on precisely identified isolates, a process that is currently impeded by the lack of taxonomic expertise (see Georgieva *et al.* [6] for detailed discussion). This highlights the need for combining molecular data with thorough morphological descriptions that will allow species delimitation and recognition in future studies. This study addresses the application of an integrative taxonomic approach to the cryptic species diversity of *Diplostomum* spp. in natural lymnaeid snail populations in Europe with a focus on the ‘*D. mergi*’ species complex. Detailed morphological and molecular data gathered in an extensive sampling of four lymnaeid species in five reservoirs of the Ruhr and its tributaries provided evidence for three named species and four distinct lineages of *Diplostomum* spp. The thorough morphological descriptions of the cercariae of *D. parviventosum* Dubois, 1932, *D. pseudospathaceum*, *D. spathaceum* and of the three novel lineages of the ‘*D. mergi*’ species complex and ‘*Diplostomum* sp. Clade Q’, in association with the molecular delineation provided here, will serve as a baseline for species identification of these important parasites of snail and fish populations and thus advance further studies on the distribution of *Diplostomum* spp. in Europe.

## Methods

### Sample collection

**Fig. 1:**
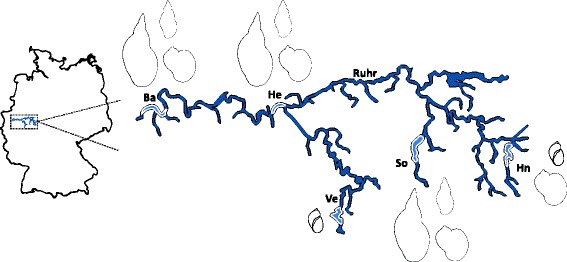
Map of the Ruhr river system in Germany with sampling sites. *Abbreviations*: Ba, Baldeneyesee; He, Hengsteysee; Hn, Hennetalsperre; So, Sorpetalsperre; Ve, Versetalsperre.  *Lymnaea stagnalis*;  *Radix auricularia*;  *Radix peregra*;  *Stagnicola palustris*

A total of 3,171 lymnaeid snails of four species [1,909 *Radix auricularia* (L.), 349 *R. peregra* (Müller), 668 *Stagnicola palustris* (Müller), 245 *Lymnaea stagnalis* (L.)] was collected and examined for trematode infections during the summer months (May to September) in 2012 and 2013. Snails were collected at several sampling sites in five reservoirs of the River Ruhr catchment area in North Rhine-Westphalia, Germany: Baldeneysee (51°24′20.08″N, 7°2′22.47″E); Hengsteysee (51°24′52.17″N, 7°27′42.55″E); Hennetalsperre (51°19′50.97″N, 8°15′46.82″E); Sorpetalsperre (51°20′15.01″N, 7°56′46.18″E); and Versetalsperre (51°10′55.71″N, 7°40′57.12″E) (see Fig. 1 and Table 1 for details). At each sampling site snails were collected randomly with hand-nets or picked by hand from sediment, stones and floating aquatic vegetation along the shore. In the laboratory all snails were measured, labelled and placed in separate beakers with lake water under a light source to stimulate cercarial emission. Snails that did not emit cercariae for several days were dissected and examined for the presence of prepatent infections. Prevalence was calculated for distinct samples (*i.e.* collected from one site within a locality on a given date) comprising more than 15 snails.

**Table 1:** Summary data for the lymnaeid snails examined/infected with *Diplostomum* spp. in five reservoirs of the River Ruhr catchment area in Germany

	Ba	He	So	Hn	Ve	Total
Total number of lymnaeid snails examined	437	1,772	357	292	313	3,171
Number of snails infected with *Diplostomum* spp.	8	66	4	–	–	78
Number of distinct samples with *Diplostomum* spp. infections	7 (0)*	29 (20)*	2 (2)*	–	–	38 (22)*

*Abbreviations*: Ba, Baldeneysee; He, Hengsteysee; Hn, Hennetalsperre; So, Sorpetalsperre; Ve, Versetalsperre

*Distinct samples with n ≥ 15 in which *Diplostomum* spp. infections were detected (number in parentheses)

### Morphological data

Cercariae were identified alive under light microscope and cercariae of *Diplostomum* spp. were identified based on the morphological descriptions and the key of Niewiadomska & Kiselienė [10]. Detailed light microscopy photographs of cercariae of *Diplostomum* spp. were taken with an Olympus UC30 digital camera on an Olympus BX51 microscope and all visible (under light microscopy) features described by Niewiadomska & Kiselienė [10] were recorded. Cercariae and/or sporocysts of all isolates were fixed in molecular grade ethanol for DNA isolation and sequencing and in cold and hot 4 % formaldehyde solution for detailed analysis of the surface morphology and body spination by scanning electron microscopy (SEM) and for obtaining measurements from fixed materials. Formalin-fixed cercariae were post-fixed in 2 % osmium tetroxide for two hours, washed in 0.1 M phosphate buffer, dehydrated through an acetone series, point-dried and sputter-coated with gold for SEM. SEM photographs were taken with a JEOL JSM-7401 F field emission scanning electron microscope. Descriptions are based on examination of live and formalin-fixed material and digital photomicrographs from both light microscopy and SEM. Measurements were taken with the program ImageJ 1.47v [11]; measurements in the descriptions are based on live specimens; measurements from fixed material are provided in the tables. All measurements in the text and tables are in micrometres and are presented as the range followed by the mean in parentheses. The following abbreviations for the metrical features were used: BL, body length; BW, maximum body width; AOL, anterior organ length; AOW, anterior organ width; VSL, ventral sucker length; VSW, ventral sucker width; TSL, tail stem length; TSW, tail stem width (at base); FL, furca length (see Additional file 1: Figure S1 for a schematic illustration of a cercaria showing the metrical features). The following relative proportions (ratios) were calculated for both live and fixed cercariae and used in addition to these measurements: VSW/AOW, ventral sucker width to anterior organ width ratio; BL/TSL, body length to tail stem length ratio; TSL/FL, tail stem length to furca length ratio.

### Molecular data

Total genomic DNA was isolated from 100–200 ethanol-fixed cercariae obtained from single snail individuals using the Chelex method (see [12] for details). Partial fragments of the barcode region of the *cox*1 mitochondrial gene were amplified via polymerase chain reaction (PCR) using Ready-To-Go PCR beads (GE Healthcare, UK) and the PCR primers Plat-diploCOX1F (5′-CGT TTR AAT TAT ACG GAT CC-3′) and Plat-diploCOX1R (5′-AGC ATA GTA ATM GCA GCA GC-3′) [13] as described in Georgieva *et al.* [6]. PCR amplifications of the ITS1-5.8S-ITS2 gene cluster were performed as above using the primers D1 (forward: 5′-AGG AAT TCC TGG TAA GTG CAA G-3′) and D2 (reverse: 5′-CGT TAC TGA GGG AAT CCT GGT-3′) [14].

PCR amplicons were purified using a QIAquick PCR purification kit (Qiagen Ltd, UK) and sequenced directly from both strands using the PCR primers (*cox*1) and the primers BD1 (forward: 5′-GTC GTA ACA AGG TTT CCG TA-3′) and BD2 (reverse: 5′-TAT GCT TAA ATT CAG CGG GT-3′) (ITS1-5.8S-ITS2; [14]) with ABI BigDye chemistry (ABI Perkin-Elmer, UK), alcohol-precipitated, and run on an ABI Prism 3130x1 automated sequencer. Contiguous sequences were assembled with MEGA v6 [15] and submitted to GenBank.

Sequences were aligned with Muscle implemented in MEGA v6. The 51 newly-generated *cox*1 sequences were aligned with reference to the amino acid translation, using the echinoderm and flatworm mitochondrial code [16] together with 28 sequences retrieved from GenBank, the latter comprising 1 − 4 representative sequences per species/lineage identified in previous studies in Europe [6, 17]; (see Additional file 2: Table S1 for details). The ITS1-5.8S-ITS2 sequences generated from selected isolates (n = 13) from the *cox*1-derived clades were aligned with 32 published sequences, representative for the species/lineages sequenced in Europe [6, 17, 18] and Canada [5, 14]. Sequences for *Tylodelphys* spp. were used as outgroups.

Distance-based neighbour-joining (NJ) and model-based Bayesian inference (BI) algorithms were used for tree reconstruction. Prior to analyses the best-fit nucleotide substitution models were selected in jModelTest 2.1.1 [19, 20]. These were the Hasegawa-Kishino-Yano model including estimates of invariant sites and among-site rate heterogeneity (HKY + I + G) for the *cox*1 dataset and the Hasegawa-Kishino-Yano model including estimates of invariant sites (HKY + I) for the ITS dataset. BI analyses were carried out with MrBayes 3.2 [21] using Markov Chain Monte Carlo (MCMC) searches on two simultaneous runs of four chains during 10^7^ generations, sampling trees every 10^3^ generations. The first 25 % of the sampled trees were discarded as with burn-in for each data set and the consensus tree topology and the nodal support were estimated from the remaining samples as posterior probability values [22]. Distance matrices (p-distance model, *i.e.* the percentage of pairwise character differences with pairwise deletion of gaps) were calculated with MEGA v6. The numbering scheme of Georgieva *et al.* [6] for the lineages of *Diplostomum* spp. was applied for consistency.

## Results

### Overview of infections with *Diplostomum* spp.

Examination of 3,171 lymnaeid snails belonging to four species revealed a total of 78 infections with *Diplostomum* spp. (overall prevalence of 2.5 %): 35 in *Radix auricularia*, 27 in *Lymnaea stagnalis* and 16 in *Stagnicola palustris*; no infections were found in *Radix peregra*. The majority of snails infected with *Diplostomum* spp. was collected in Hengsteysee, supporting the most abundant snail populations, whereas only few infected snails were found in Baldeneysee and Sorpetalsperre due to the lower snail density resulting in smaller sample size and none were recorded in either Hennetalsperre or Versetalsperre (Table 1). Prevalence of infections with *Diplostomum* spp. in distinct samples comprising more than 15 snails ranged from 1.0 to 16.7 % (Table 2). The data in Table 2 also reveal the high diversity of *Diplostomum* spp. in Hengsteysee.

**Table 2:** Prevalence of *Diplostomum* spp. in the distinct samples (n ≥ 15) of the three lymnaeid snail hosts examined

Reservoir	Hengsteysee			Sorpetalsperre
Snail species	*R. auricularia*	*L. stagnalis*	*S. palustris*	*R. auricularia*
*D. parviventosum*	3.1–7.1 (2)			
‘*D. mergi* Lineage 2’	2.1–6.7 (4)			2.2–10.7 (2)
‘*D. mergi* Lineage 3’	1.0–3.1 (3)			
*D. mergi* Lineage 4	1.0 (1)			
‘*Diplostomum* sp. Clade Q’	3.6 (1)			
*D. spathaceum*	2.1–4.1 (4)			
*D. pseudospathaceum*		3.7–16.7 (6)	1.0–8.7 (5)	

Prevalence is calculated for sample size n ≥ 15 only; the number of samples is given in parentheses

Using the key and descriptions in Niewiadomska & Kiselienė [10] morphological identification of cercariae to the species level was achieved for two species, *D. parviventosum* ex *R. auricularia* and *D. pseudospathaceum* ex *L. stagnalis.* Subsamples of the remaining isolates were subjected to molecular identification based on sequence data [6, 17].

### Molecular analyses

**Fig. 2:**
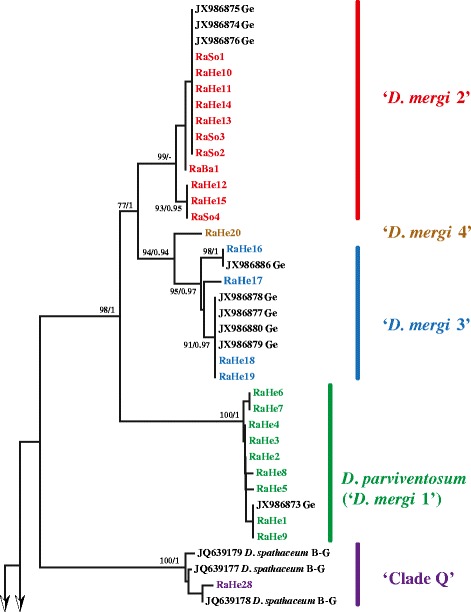
Neighbour-joining (NJ) phylogram for *Diplostomum* spp. reconstructed using 51 newly-generated and 28 *cox*1 sequences retrieved from GenBank. Nodal support from NJ and Bayesian Inference (BI) analyses indicated as NJ/BI. The scale bar indicates the expected number of substitutions per site. The newly-sequenced isolates are coded as in Table 3. Sequence identification is as in GenBank, followed by a letter: B-G, Behrmann-Godel [17]; Ge, Georgieva *et al.* [6]

**Fig. 3:**
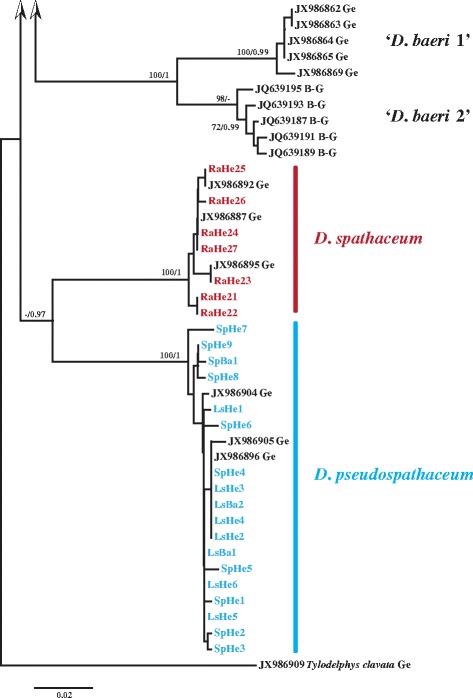
Neighbour-joining (NJ) phylogram for *Diplostomum* spp. reconstructed using 51 newly-generated and 28 *cox*1 sequences retrieved from GenBank; continuation of Fig. 2. Nodal support from NJ and Bayesian Inference (BI) analyses indicated as NJ/BI. The scale bar indicates the expected number of substitutions per site. Codes for the newly-sequenced isolates are provided in Table 3. Sequence identification is as in GenBank, followed by a letter: B-G, Behrmann-Godel [17]; Ge, Georgieva *et al.* [6]

Partial *cox*1 sequences were obtained for most of the isolates (51; 65 %) collected in Hengsteysee and Sorpetalsperre (see Table 3 for details). Both NJ and BI analyses of the *cox*1 dataset (410 nt) recovered eight reciprocally monophyletic lineages (Figs. 2, 3). The predominant part of the isolates ex *R. auricularia* (n = 25; 76 %) clustered with the isolates comprising ‘*D. mergi*’ species complex *sensu* Georgieva *et al.* [6] thus expanding substantially the content of the three lineages identified by these authors (Fig. 2). Nine isolates identified here as *D. parviventosum* based on morphology formed a strongly supported lineage (‘*D. mergi* Lineage 1’) together with the single isolate ex *R. auricularia* (JX986873) of Georgieva *et al.* [6], 11 further isolates clustered together with the three isolates ex *R. auricularia* (JX986874–JX986876) (‘*D. mergi* Lineage 2’ *sensu* Georgieva *et al.* [6]) and four isolates clustered with five metacercarial isolates ex *Salmo trutta fario* L. and *Gobio gobio* L. of the ‘*D. mergi* Lineage 3’ *sensu* Georgieva *et al.* [6]; this lineage was joined by a single isolate (RaHe20; further referred to as ‘*D. mergi* Lineage 4’). The remaining isolates ex *R. auricularia* joined two additional strongly supported lineages: *D. spathaceum* (7 isolates) and ‘Clade Q’ *sensu* Georgieva *et al.* [6] (one isolate) represented by one cercarial isolate ex *R. auricularia* (RA97) and two metacercarial isolates ex *R. rutilus* (RR43 and RR45) from Lake Constance, all reported as *D. spathaceum* (see Behrmann-Godel [17]) but annotated as *D. mergi* in the GenBank. All isolates ex *L. stagnalis* and *S. palustris* were identified as and clustered with isolates of *D. pseudospathaceum* (Fig. 3).

**Table 3:** Summary data for 51 isolates of *Diplostomum* spp. used for generation of the *cox*1 and ITS1-5.8S-ITS2 sequences

Species	Isolate	Snail host	Reservoir	GenBank accession number (*cox*1/ITS1-5.8S-ITS2)
*D. parviventosum*	RaHe1	*R. auricularia*	Hengsteysee	KR149504
*D. parviventosum*	RaHe2	*R. auricularia*	Hengsteysee	KR149505
*D. parviventosum*	RaHe3	*R. auricularia*	Hengsteysee	KR149506
*D. parviventosum*	RaHe4	*R. auricularia*	Hengsteysee	KR149507
*D. parviventosum*	RaHe5	*R. auricularia*	Hengsteysee	KR149508
*D. parviventosum*	RaHe6	*R. auricularia*	Hengsteysee	KR149509/KR149490
*D. parviventosum*	RaHe7	*R. auricularia*	Hengsteysee	KR149510
*D. parviventosum*	RaHe8	*R. auricularia*	Hengsteysee	KR149511/KR149491
*D. parviventosum*	RaHe9	*R. auricularia*	Hengsteysee	KR149512/KR149492
‘*D. mergi* Lineage 2’	RaBa1	*R. auricularia*	Baldeneysee	KR149513/KR149493
‘*D. mergi* Lineage 2’	RaHe10	*R. auricularia*	Hengsteysee	KR149514
‘*D. mergi* Lineage 2’	RaHe11	*R. auricularia*	Hengsteysee	KR149515
‘*D. mergi* Lineage 2’	RaHe12	*R. auricularia*	Hengsteysee	KR149516
‘*D. mergi* Lineage 2’	RaHe13	*R. auricularia*	Hengsteysee	KR149517
‘*D. mergi* Lineage 2’	RaHe14	*R. auricularia*	Hengsteysee	KR149518
‘*D. mergi* Lineage 2’	RaHe15	*R. auricularia*	Hengsteysee	KR149519/KR149494
‘*D. mergi* Lineage 2’	RaSo1	*R. auricularia*	Sorpetalsperre	KR149520
‘*D. mergi* Lineage 2’	RaSo2	*R. auricularia*	Sorpetalsperre	KR149521/KR149495
‘*D. mergi* Lineage 2’	RaSo3	*R. auricularia*	Sorpetalsperre	KR149522
‘*D. mergi* Lineage 2’	RaSo4	*R. auricularia*	Sorpetalsperre	KR149523
‘*D. mergi* Lineage 3’	RaHe16	*R. auricularia*	Hengsteysee	KR149524/KR149496
‘*D. mergi* Lineage 3’	RaHe17	*R. auricularia*	Hengsteysee	KR149525/KR149497
‘*D. mergi* Lineage 3’	RaHe18	*R. auricularia*	Hengsteysee	KR149526/KR149498
‘*D. mergi* Lineage 3’	RaHe19	*R. auricularia*	Hengsteysee	KR149527
*D. mergi* Lineage 4	RaHe20	*R. auricularia*	Hengsteysee	KR149528/KR149499
*D. pseudospathaceum*	LsBa1	*L. stagnalis*	Baldeneysee	KR149529
*D. pseudospathaceum*	LsBa2	*L. stagnalis*	Baldeneysee	KR149530
*D. pseudospathaceum*	LsHe1	*L. stagnalis*	Hengsteysee	KR149531
*D. pseudospathaceum*	LsHe2	*L. stagnalis*	Hengsteysee	KR149532/KR149500
*D. pseudospathaceum*	LsHe3	*L. stagnalis*	Hengsteysee	KR149533/KR149501
*D. pseudospathaceum*	LsHe4	*L. stagnalis*	Hengsteysee	KR149534
*D. pseudospathaceum*	LsHe5	*L. stagnalis*	Hengsteysee	KR149535
*D. pseudospathaceum*	LsHe6	*L. stagnalis*	Hengsteysee	KR149536
*D. pseudospathaceum*	SpBa1	*S. palustris*	Baldeneysee	KR149537
*D. pseudospathaceum*	SpHe1	*S. palustris*	Hengsteysee	KR149538
*D. pseudospathaceum*	SpHe2	*S. palustris*	Hengsteysee	KR149539
*D. pseudospathaceum*	SpHe3	*S. palustris*	Hengsteysee	KR149540
*D. pseudospathaceum*	SpHe4	*S. palustris*	Hengsteysee	KR149541
*D. pseudospathaceum*	SpHe5	*S. palustris*	Hengsteysee	KR149542
*D. pseudospathaceum*	SpHe6	*S. palustris*	Hengsteysee	KR149543
*D. pseudospathaceum*	SpHe7	*S. palustris*	Hengsteysee	KR149544
*D. pseudospathaceum*	SpHe8	*S. palustris*	Hengsteysee	KR149545
*D. pseudospathaceum*	SpHe9	*S. palustris*	Hengsteysee	KR149546
*D. spathaceum*	RaHe21	*R. auricularia*	Hengsteysee	KR149547
*D. spathaceum*	RaHe22	*R. auricularia*	Hengsteysee	KR149548
*D. spathaceum*	RaHe23	*R. auricularia*	Hengsteysee	KR149549
*D. spathaceum*	RaHe24	*R. auricularia*	Hengsteysee	KR149550
*D. spathaceum*	RaHe25	*R. auricularia*	Hengsteysee	KR149551/KR149502
*D. spathaceum*	RaHe26	*R. auricularia*	Hengsteysee	KR149552
*D. spathaceum*	RaHe27	*R. auricularia*	Hengsteysee	KR149553
‘*Diplostomum* sp. Clade Q’	RaHe28	*R. auricularia*	Hengsteysee	KR149554/KR149503

**Fig. 4:**
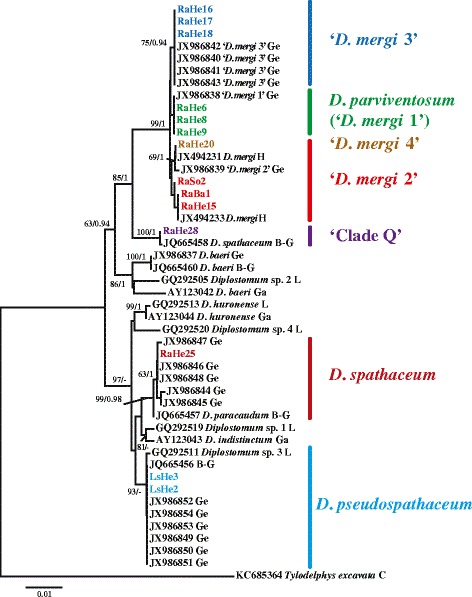
Neighbour-joining (NJ) phylogram for *Diplostomum* spp. reconstructed using 13 newly-generated and 32 ITS1-5.8S-ITS2 sequences retrieved from GenBank. Outgroup: *Tylodelphys excavata*. Nodal support from NJ and Bayesian Inference (BI) analyses are indicated as NJ/BI. The scale bar indicates the expected number of substitutions per site. Codes for the newly-sequenced isolates are provided in Table 3. Sequence identification is as in GenBank, followed by a letter: B-G, Behrmann-Godel [17]; Ge, Georgieva *et al.* [6]; Ga, Galazzo *et al.* [14]; H, Haarder *et al.* [18]; L, Locke *et al.* [5]

The mean intraspecific divergence within the *cox*1 dataset examined ranged between 0.30 and 0.95 %, *i.e.* below the range for mean divergence in the interspecific comparisons (4.3–14.7 %) (Table 4). The lowest value was obtained for the sister lineages 2 and 3 of the ‘*D. mergi*’ complex. The single isolate of *D. mergi* Lineage 4 exhibited lower divergence values in comparisons with isolates of Lineages 2 and 3 (3.7–3.9 and 2.4 %, respectively). A total of 13 ITS1-5.8S-ITS2 sequences (975 nt) was generated from isolates sub-sampled within the seven *cox*1 lineages of newly-collected *Diplostomum* spp. The analysis of the ITS data supported the molecular identification of these isolates from *cox*1 gene trees except for *D. mergi* Lineage 4 which clustered within the isolates of ‘*D. mergi* Lineage 2’ (Fig. 4).

**Table 4:** Mean divergence (uncorrected p-distance in %) estimated for the *cox*1 sequence pairs within- (diagonal) and among species and lineages of *Diplostomum*

		1	2	3	4	5	6	7	8
1	‘*Diplostomum* sp. Clade Q’	0.56							
2	*D. baeri* (trout) 1	13.5	0.44						
3	*D. baeri* (perch) 2	13.3	6.5	0.95					
4	D. parviventosum*	11.1	13.9	13.0	0.30				
5	‘*D. mergi* Lineage 2’	10.9	13.8	13.8	6.7	0.30			
6	‘*D. mergi* Lineage 3’	11.0	14.4	14.7	6.9	4.3	0.65		
7	*D. pseudospathaceum*	12.0	14.7	13.2	13.9	12.0	12.9	0.66	
8	*D. spathaceum*	12.1	14.6	12.4	11.2	10.7	11.6	10.0	0.53

*‘*D. mergi* Lineage 1’ of Georgieva *et al.* [6]

Detailed morphological assessment of the isolates following the identification of independent evolutionary lineages confirmed their distinct status. Using the new set of morphological characters defined for each lineage, all isolates were assigned to lineage. Taken together, the results from the molecular and morphological analyses suggest that isolates sampled in lymnaeid snails from Germany represent three named species and four distinct lineages of *Diplostomum* spp. Descriptions of the cercariae of *D. parviventosum*, *D. pseudospathaceum*, *D. spathaceum* and of the three novel lineages of the ‘*D. mergi*’ species complex plus ‘*Diplostomum* sp. Clade Q’ are provided below.

### Descriptions of the cercariae of *Diplostomum* spp. based on the molecular voucher material

#### *Diplostomum parviventosum* Dubois, 1932

***First intermediate host***: *Radix auricularia* (Linnaeus).

***Locality***: Hengsteysee, Germany.

**Fig. 5:**
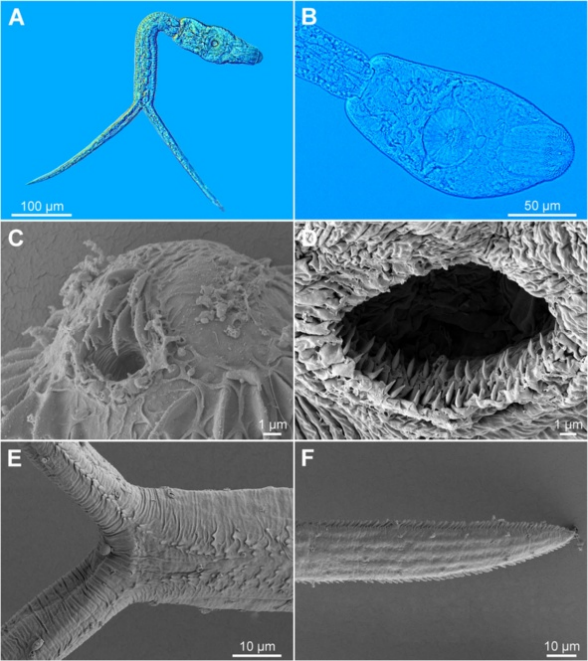
Cercaria of *Diplostomum parviventosum* ex *Radix auricularia* (light and scanning electron microscopy, SEM). **a**, Resting position; **b**, Body; **c**, Anterior organ, apical view (SEM); **d**, Ventral sucker (SEM); **e**, Tail stem and furcae (SEM); **f**, Furcae (SEM)

[Figure 5 and Additional file 3: Figure S2A, Additional file 4: Figure S3A, Additional file 5: Figure S4A. Measurements of formalin-fixed specimens are provided in Table 5.] Body elongate-oval, 127 − 164 × 53 − 86 (146 × 62), shorter than tail stem [BL/TSL = 0.7 − 0.9 (0.8)], with aggregations of yellow pigment in parenchyma on both sides of terminal organ and around ventral sucker (Fig. 5a, b). Anterior organ elongate-oval, with posterior margin reaching to mid-length of forebody, 42 − 67 × 23 − 40 (51 × 32). Ventral sucker spherical, small, somewhat post-equatorial, 31 − 44 × 30 − 48 (37 × 37), with fine undulating membrane (3 high) (Fig. 5d); width slightly exceeds width of anterior organ [VSW/AOW = 1.1 − 1.3 (1.2)]. Penetration gland-cells 2 pairs, relatively small, with fine granular content, posterior to ventral sucker, overlap caeca partially, posterior pair not reaching extremities of caeca; ducts open antero-laterally to mouth. Tail stem 148 − 208 (173) long, 27 − 40 (33) wide at base, slightly shorter than furcae [TSL/FL = 0.7 − 1.0 (0.8)], contains 10 − 12 pairs of caudal bodies, irregularly shaped but with smooth contours. Furcae 199 − 234 (216) long, with fish-fin like fin-folds.

**Table 5:** Comparative metrical data for cercariae of the *Diplostomum* ‘*mergi*’ species complex

Species	*D. mergi*	‘*D. mergi* Lineage 2’	‘*D. mergi* Lineage 3’	*D. parviventosum*	
Source	Niewiadomska & Kiselienė [10]	Present study	Present study	Niewiadomska & Kiselienė [10]	Present study
Fixation method	Heat-killed in water	Formalin	Formalin	Heat-killed in water	Formalin
BL	161–204 (182)	157–179 (168)	171–191 (184)	162–185 (176)	115–151 (134)
BW	51–68 (59)	62–67 (64)	44–54 (49)	50–70 (55)	50–61 (54)
AOL	51–68 (58)	43–53 (49)	42–59 (48)	55–60 (56)	43–53 (47)
AOW	25–30 (28)	24–36 (33)	29–35 (32)	29–32 (31)	26–33 (30)
VSL	34–51 (44)	43–49 (46)	28–33 (31)	34–38 (36)	29–39 (33)
VSW	34–51 (44)	50–56 (53)	28–35 (32)	34–37 (36)	30–46 (37)
TSL	187–229 (207)	146–180 (168)	196–224 (212)	203–242 (228)	153–176 (165)
TSW	34–43 (36)	27–32 (29)	30–36 (33)	31–40 (35)	26–36 (30)
FL	187–229 (210)	203–227 (216)	210–245 (225)	191–218 (206)	178–217 (204)
VSW/AOW	(1.6)	1.5–2.1 (1.6)	0.9–1.1 (1.0)	(1.1)	1.0–1.4 (1.2)
BL/TSL	(0.9)	1.0–1.1 (1.0)	0.8–1.0 (0.9)	(0.8)	0.7–0.9 (0.8)
TSL/FL	(1.0)	0.7–0.8 (0.8)	0.9–1.0 (0.9)	(1.1)	0.8–0.9 (0.8)

Data are presented as the range followed by the mean in parentheses. See Methods and Additional file 1: Figure S1 for description and illustration of the metrical features

Body armature: Pre-oral spines arranged in median group of 6 − 7 spines in 3 rows; lateral groups absent (Fig. 5c, Additional file 3: Figure S2A). Post-oral spines more robust than spines on body, in 7 − 8 alternate rows; rows 1 − 2 with median interruption; first 4 spines in row 1 on both sides of median interruption largest; spines in row 1 larger than remaining spines, all of similar size. Wide zone of smaller, less dense, irregularly dispersed spines present posterior to post-oral spines, followed by narrow spineless area and 11 transverse rows of spines extending to mid-level of ventral sucker. Rows 1 − 4 complete (*i.e.* encircling body); rows 5 − 8 discontinuous dorsally; rows 9 − 11 discontinuous ventrally and dorsally; rows 1 and 10 with additional spines laterally. Two ventro-lateral fields of smaller spines (0.8–1.2) present posterior to ventral sucker; fields converge close to posterior extremity of body. Ventral sucker armed with 2 rows of spines (*c.*40 per row; range 77 − 87; mean 81) (Fig. 5d, Additional file 4: Figure S3A). Tail stem and furcae with scale-like spines (Fig. 5e); spines on tail stem in 4 medio-lateral bands (2 ventral and 2 dorsal), each consisting of 2 − 3 scale-like spines, increasing in size posteriorly (0.6 − 2), plus a median row of minute spines; bands continue along furcal margins as rows of 2 spines anteriorly and single spine posteriorly; all spines on furcae enveloped by tegumental membrane forming fish-fin like fin-fold (Fig. 5f, Additional file 5: Figure S4A).

Resting position: Tail stem bent at < 90° (45–67°).

#### ‘*Diplostomum mergi* Lineage 2’ of Georgieva *et al.* [6]

***First intermediate host***: *Radix auricularia* (Linnaeus).

***Locality***: Hengsteysee, Sorpetalsperre, Germany.

**Fig. 6:**
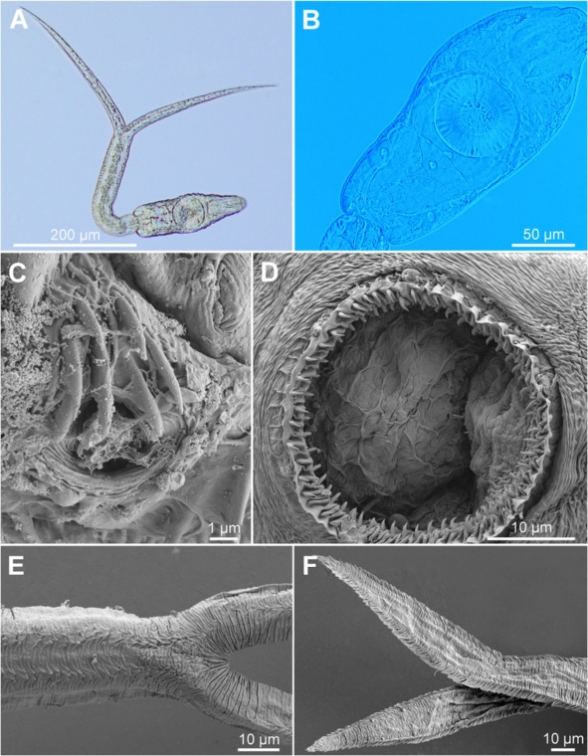
Cercaria of ‘*Diplostomum mergi* Lineage 2’ of Georgieva *et al.* [6]) ex *Radix auricularia* (light and scanning electron microscopy, SEM). **a**, Resting position; **b**, Body; **c**, Anterior organ, apical view (SEM); **d**, Ventral sucker (SEM); **e**, Tail stem and furcae (SEM); **f**, Furcae (SEM)

[Figure 6 and Additional file 3: Figure S2C, Additional file 4: Figure S3C, Additional file 5: Figure S4C. Measurements from formalin-fixed specimens are provided in Table 5.] Body elongate-oval, 154 − 179 × 60 − 66 (167 × 63), slightly shorter than tail stem [BL/TSL = 0.8 − 0.9 (0.9)], with aggregations of yellow pigment in parenchyma on both sides of terminal organ and around ventral sucker, most prominently anterior to ventral sucker (Fig. 6a, b). Anterior organ elongate-oval, with posterior margin reaching to mid-length of forebody, 50 − 62 × 26 − 30 (56 × 28). Ventral sucker subspherical, large, somewhat post-equatorial, 47 − 53 × 42 − 52 (50 × 47), with fine undulating membrane (3 − 4 high) (Fig. 6d); width exceeds width of anterior organ [VSW/AOW = 1.5 − 2.0 (1.7)]. Penetration gland-cells 2 pairs, large, with fine granular content, posterior to ventral sucker, overlap caeca partially, posterior pair not reaching extremities of caeca; ducts open antero-laterally to mouth. Tail stem 185 − 207 (195) long, 31 − 36 (33) wide at base, shorter than furcae [TSL/FL = 0.8 − 0.9 (0.8)], contains 36 − 40 caudal bodies; individual caudal bodies with smooth contours. Furcae 221 − 273 (247) long, with fish-fin like fin-folds.

Body armature: Pre-oral spines arranged in median group of 5 − 6 spines in 2 rows with one median spine very large (Fig. 6c, Additional file 3: Figure S2C); one additional very small spine may be present; lateral groups absent. Post-oral spines more robust than spines on body, in 11 alternate rows (one additional median row may be present); rows 1 − 2 with median interruption; first 4 spines in row 1 and first 3 spines in row 2 on both sides of median interruption largest; spines in row 1 larger than remaining spines, all of similar size. Wide zone of smaller, less dense, irregularly dispersed spines present posterior to post-oral spines, followed by narrow spineless area and 10 transverse rows of spines extending to mid-level of ventral sucker; row 10 with smaller and fewer spines. Rows 1 − 5 complete (*i.e.* encircling body); rows 6 − 10 discontinuous ventrally and dorsally; rows 1 − 4 with additional spines laterally; rows 5 and 6 with few additional spines laterally. Two ventro-lateral fields of smaller spines (1.0–1.5) present posterior to ventral sucker; fields converge close to posterior extremity of body. Ventral sucker armed with 2 rows of spines (*c.*57 per row; range 110 − 120; mean 114) (Fig. 6d, Additional file 4: Figure S3C). Tail stem and furcae with scale-like spines (Fig. 6e); spines on tail stem in 4 medio-lateral bands (2 ventral and 2 dorsal), starting from second quarter of tail, each consisting of 2 − 3 scale-like spines, increasing in size posteriorly (0.6 − 2.5); bands continue along furcal margins as rows of 2 spines anteriorly and 1 spine posteriorly; all spines on furcae enveloped by tegumental membrane forming fish-fin like fin-fold (Fig. 6f, Additional file 5: Figure S4C).

Resting position: Tail stem bent at < 90° (64 − 85°).

#### ‘*Diplostomum mergi* Lineage 3’ of Georgieva *et al.* [6]

***First intermediate host***: *Radix auricularia* (Linnaeus).

***Locality***: Hengsteysee, Germany.

**Fig. 7:**
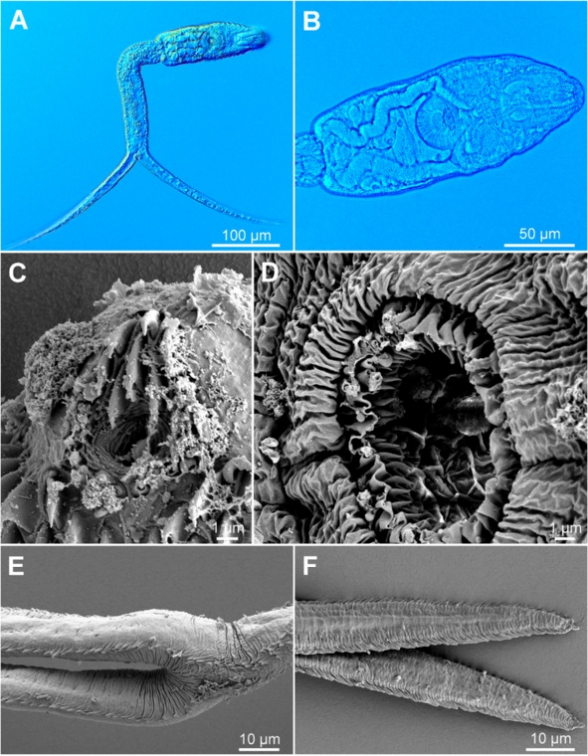
Cercaria of ‘*Diplostomum mergi* Lineage 3’ of Georgieva *et al.* [6] ex *Radix auricularia* (light and scanning electron microscopy, SEM). **a**, Resting position; **b**, Body; **c**, Anterior organ, apical view (SEM); **d**, Ventral sucker (SEM); **e**, Tail stem and furcae (SEM); **f**, Furcae (SEM)

[Figure 7 and Additional file 3: Figure S2D, Additional file 4: Figure S3D, Additional file 5: Figure S4D. Measurements of formalin-fixed specimens are provided in Table 5.] Body elongate-oval, 158 − 169 × 50 − 57 (162 × 53), slightly shorter than tail stem [BL/TSL = 0.9 − 1.0 (0.9)], with aggregations of yellow pigment in parenchyma on both sides of ventral sucker (Fig. 7a, b). Anterior organ elongate-oval, with posterior margin reaching to mid-length of forebody, 50 − 57 × 26 − 29 (54 × 28). Ventral sucker spherical, small, somewhat post-equatorial, 29 − 34 × 28 − 32 (31 × 31), with fine undulating membrane (2 high) (Fig. 7d); width slightly exceeds width of anterior organ [VSW/AOW = 1.0 − 1.2 (1.1)]. Penetration gland-cells 2 pairs, medium-sized, with fine granular content, posterior to ventral sucker, overlap caeca partially, posterior pair not reaching extremities of caeca, ducts open antero-laterally to mouth. Tail stem 167 − 197 (179) long, 31 − 33 (32) wide at base, shorter than furcae [TSL/FL = 0.7 − 0.8 (0.8)], contains numerous (impossible to count) caudal bodies with incised contours. Furcae 215 − 240 (227) long, with fish-fin like fin-folds.

Body armature: Pre-oral spines arranged in median group of 7 spines in 3 rows with one median spine slightly larger (Fig 7c, Additional file 3: Figure S2D); lateral groups absent. Post-oral spines more robust than spines on body, in 10 alternate rows; rows 1 − 2 with a median interruption; first 4 spines in row 1 on both sides of median interruption largest; spines in rows 1 − 2 larger than remaining spines, all of similar size. Wide zone of smaller, less dense, irregularly dispersed spines present posterior to post-oral spines, followed by narrow spineless area and 11 transverse rows of spines extending to mid-level of ventral sucker; row 11 with smaller spines. Rows 1 − 3 complete (*i.e.* encircling body); rows 4 − 11 discontinuous ventrally and dorsally; row 1 doubled; rows 2 − 3 with additional spines laterally. Two ventro-lateral non-converging fields of smaller spines (0.7–1.0) present posterior to ventral sucker. Ventral sucker armed with 2 rows of spines (*c.*45 per row; range 90 − 92; mean 90) (Fig. 7d, Additional file 4: Figure S3D). Tail stem and furcae with scale-like spines (Fig. 7e); spines on tail stem in 4 medio-lateral bands (2 ventral and 2 dorsal), each consisting of 2 − 3 scale-like spines, increasing in size posteriorly (0.8 − 3.0); bands continue along furcal margins as rows of 2 spines anteriorly and 1 spine posteriorly; all spines on furcae enveloped by tegumental membrane forming fin-fold (Fig. 7f, Additional file 5: Figure S4D).

Resting position: Tail stem bent at ≤ 90° (77 − 91°).

#### *Diplostomum mergi* Lineage 4

***First intermediate host***: *Radix auricularia* (Linnaeus).

***Locality***: Hengsteysee, Germany.

**Fig. 8:**
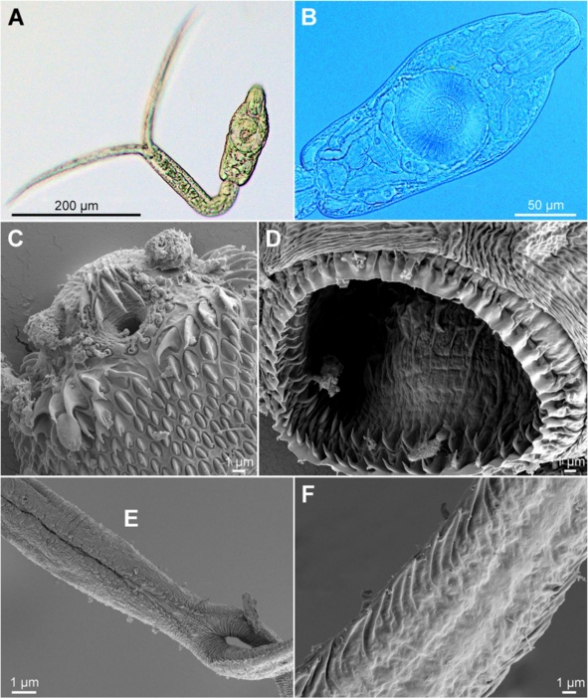
Cercaria of *Diplostomum mergi* Lineage 4 ex *Radix auricularia* (light and scanning electron microscopy, SEM). **a**, Resting position; **b**, Body; **c**, Anterior organ, apical view (SEM); **d**, Ventral sucker (SEM); **e**, Tail stem and furcae (SEM); **f**, Furcae (SEM)

[Figure 8 and Additional file 3: Figure S2B, Additional file 4: Figure S3B, Additional file 5: Figure S4B.] Body elongate-oval, 180 − 186 × 94 − 115 (184 × 105), shorter than tail stem (BL/TSL = 0.7), with aggregations of yellow pigment in parenchyma on both sides of terminal organ and around ventral sucker (Fig. 8a, b). Anterior organ elongate-oval, with posterior margin reaching to mid-length of forebody, 54 − 74 × 38 − 51 (63 × 44). Ventral sucker transversely oval, large, somewhat post-equatorial, 58 − 79 × 65 − 78 (63 × 71), with fine undulating membrane (3 − 5 high) (Fig. 8d); width exceeds width of anterior organ [VSW/AOW = 1.5 − 1.7 (1.6)]. Penetration gland-cells 3 pairs (anterior pair small, posterior pairs large), with fine granular content, posterior to ventral sucker, overlap caeca partially, posterior pair not reaching extremities of caeca; ducts open antero-laterally to mouth. Tail stem 261 − 263 (262) long, 41 − 47 (43) wide at base, of equal length as furcae (TSL/FL = 1.0), with 36 − 40 caudal bodies; individual caudal bodies irregularly shaped with smooth contours. Furcae 269 long, with fish-fin like fin-folds.

Body armature: Pre-oral spines arranged in median group of 7 spines in 3 rows with one median spine very large (Fig. 8c, Additional file 3: Figure S2B); lateral groups absent. Post-oral spines more robust than spines on body, in 11 alternate rows; rows 1 − 2 with median interruption, rows 10 − 11 interrupted dorsally; first 4 spines in row 1 and first 3 spines in row 2 on both sides of median interruption largest; spines in first 2 rows larger than remaining spines, all of similar size. Wide zone of smaller, less dense, irregularly dispersed spines present posterior to post-oral spines, followed by narrow spineless area and 10 transverse rows of spines extending to mid-level of ventral sucker. Rows 1 − 4 complete (*i.e.* encircling body); rows 5 − 7 discontinuous dorsally; rows 8 − 10 discontinuous ventrally and dorsally. Two ventro-lateral fields of smaller spines (0.5–1.0) present posterior to ventral sucker; fields converge close to posterior extremity of body. Ventral sucker armed with 2 rows of spines (*c.*56 per row; range 112 − 114; mean 113) (Fig. 8d, Additional file 4: Figure S3B). Tail stem and furcae with scale-like spines (Fig. 8e); spines on tail stem in 4 medio-lateral bands (2 ventral and 2 dorsal), each consisting of 1 − 2 scale-like spines, increasing in size posteriorly (1 − 3); bands continue along furcal margins as single rows of spines; all spines on furcae enveloped by tegumental membrane forming fish-fin like fin-fold (Fig. 8f, Additional file 5: Figure S4B).

Resting position: Tail stem bent at < 90° (66°).

#### *‘Diplostomum* sp. Clade Q’ of Georgieva *et al.* [6]

***First intermediate host***: *Radix auricularia* (Linnaeus).

***Locality***: Hengsteysee, Germany.

**Fig. 9:**
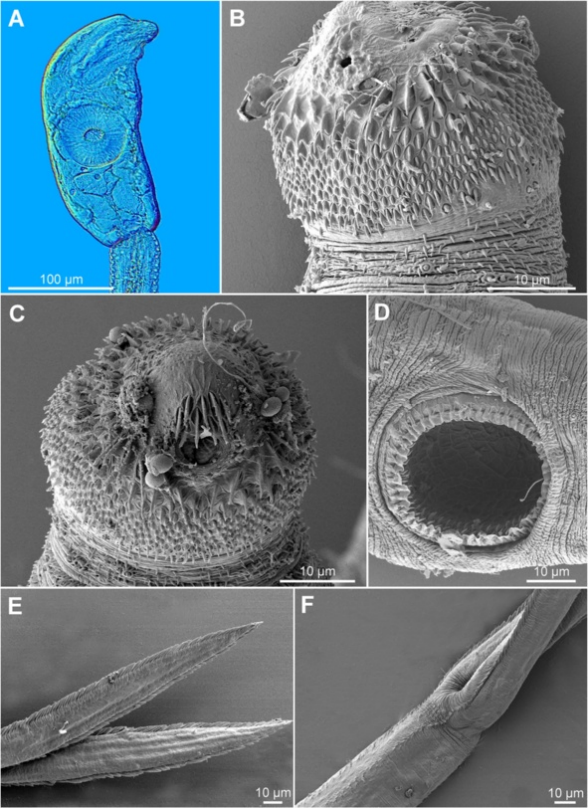
Cercaria of ‘*Diplostomum* sp. Clade Q’ of Georgieva *et al.* [6] ex *Radix auricularia* (light and scanning electron microscopy, SEM). **a**, Body; **b**, Anterior organ, lateral view (SEM); **c**, Anterior organ, apical view (SEM); **d**, Ventral sucker (SEM); **e**, Furcae (SEM); **f**, Tail stem and furcae (SEM)

[Figure 9 and Additional file 4: Figure S3E, Additional file 5: Figure S4E.] Body elongate-oval, 215 − 239 × 87 − 101 (224 × 96), shorter than tail stem (BL/TSL = 0.8), with aggregations of yellow pigment in parenchyma on both sides of terminal organ, around ventral sucker (Fig. 9a) and in furcae. Anterior organ elongate-oval, with posterior margin reaching to mid-length of forebody, 70 − 88 × 46 − 48 (80 × 47). Ventral sucker transversely oval, large, somewhat post-equatorial, 51 − 60 × 57 − 70 (56 × 65), with fine undulating membrane (5 high) (Fig. 9d); width exceeds width of anterior organ [VSW/AOW = 1.2 − 1.4 (1.3)]. Penetration gland-cells 2 pairs, large, with fine granular content, posterior to ventral sucker, overlap caeca partially, posterior pair not reaching extremities of caeca; ducts open antero-laterally to mouth. Tail stem 266 long, 43 − 48 (46) wide at base, of equal length to furcae (TSL/FL = 1.0), contains 10 pairs of caudal bodies with incised contours. Furcae 280 long, with fish-fin like fin-folds.

Body armature: Pre-oral spines arranged in median group of 9 spines in 3 rows (Fig. 9c); lateral groups absent. Post-oral spines more robust than spines on body, in 12 alternate rows; first row with median interruption, rows 11–12 interrupted laterally; first 5 spines in row 1 on both sides of median interruption largest; remaining spines of different sizes, spines in rows 1–4 distinctly larger than those in remaining rows, spines in rows 5–10 small, spines in rows 10–12 medium-sized (Fig. 9b). Wide zone of smaller, less dense, irregularly dispersed spines present posterior to post-oral spines (spines sparser dorsally), followed by narrow spineless area and 10 transverse rows of spines extending to mid-level of ventral sucker. Rows 1 − 4 complete (*i.e.* encircling body); rows 5 − 10 discontinuous ventrally and dorsally; row 1 doubled, rows 2 − 3 with additional spines laterally. Two ventro-lateral non-converging fields of smaller spines (0.7–1.3) present posterior to ventral sucker. Ventral sucker armed with 2 rows of spines (*c.*57 per row; range 112 − 116; mean 114) (Fig. 9d, Additional file 4: Figure S3E). Tail stem and furcae with scale-like spines (Fig. 9f); spines on tail stem in 4 medio-lateral bands (2 ventral and 2 dorsal), each consisting of 1 − 2 scale-like spines anteriorly and of 3 spines posteriorly close to bifurcation; spines increase in size posteriorly (0.6 − 3.0); bands continue along furcal margins as single rows of spines; all spines on furcae enveloped by tegumental membrane forming fish-fin like fin-folds (Fig. 9e, Additional file 5: Figure S4E).

Resting position not observed.

#### *Diplostomum spathaceum* (Rudolphi, 1819)

***First intermediate host***: *Radix auricularia* (Linnaeus).

***Locality***: Hengsteysee, Germany.

**Fig. 10:**
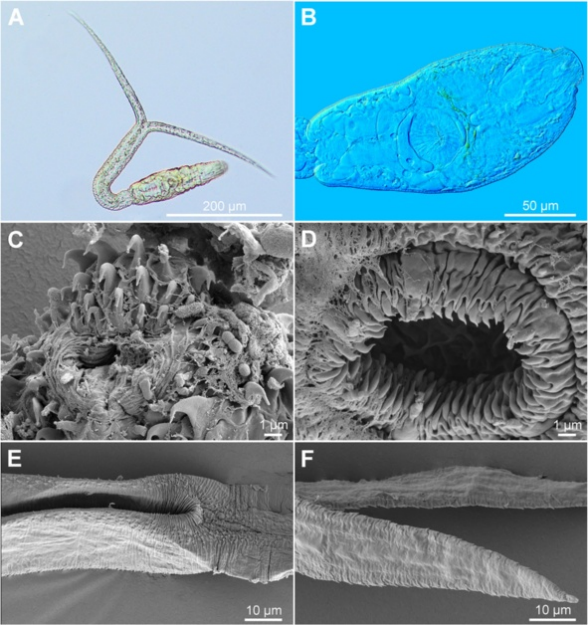
Cercaria of *Diplostomum spathaceum* ex *Radix auricularia* (light and scanning electron microscopy, SEM). **a**, Resting position; **b**, Body; **c**, Anterior organ, apical view (SEM); **d**, Ventral sucker (SEM); **e**, Tail stem and furcae (SEM); **f**, Furcae (SEM)

[Figure 10 and Additional file 3: Figure S2G, Additional file 4: Figure S3G, Additional file 5: Figure S4G. Measurements of formalin-fixed specimens are provided in Table 6.] Body elongate-oval, 159 − 178 × 46 − 60 (172 × 51), shorter than tail stem [BL/TSL = 0.7 − 0.9 (0.8)], with aggregations of yellow pigment in parenchyma concentrated on both sides of anterior organ and above ventral sucker (Fig. 10a, b). Anterior organ elongate-oval, with posterior margin reaching to mid-length of forebody, 39 − 56 × 24 − 28 (50 × 26). Ventral sucker spherical, small, somewhat post-equatorial, 26 − 35 × 27 − 33 (30 × 30), with fine undulating membrane (2 − 3.5 high) (Fig. 10d); width slightly exceeds width of anterior organ [VSW/AOW = 1.0 − 1.3 (1.1)]. Penetration gland-cells 2 pairs, large, with fine granular content, posterior to ventral sucker, overlap caeca partially, posterior pair not reaching extremities of caeca. Tail stem 197 − 233 (219) long, 29 − 32 (31) wide at base, shorter than furcae [TSL/FL = 0.8 − 0.9 (0.8)], contains 56 − 60 caudal bodies; individual caudal bodies irregularly shaped with both incised and smooth contours. Furcae 250 − 267 (260) long, without fin-fold.

**Table 6:** Comparative metrical data for cercariae of *Diplostomum pseudospathaceum*, *D. spathaceum* and *D. paracaudum*

Species	*D. pseudospathaceum*		*D. spathaceum*	*D. paracaudum*	*D. spathaceum*
Source	Niewiadomska [27]; Niewiadomska & Kiselienė [10]	Present study	Niewiadomska [27]; Niewiadomska & Kiselienė [10]	Niewiadomska [28]; Niewiadomska & Kiselienė [10]	Present study
Fixation method	Heat-killed in water	Formalin	Heat-killed in water	Heat-killed in water	Formalin
BL	170–199 (185)	150–172 (162)	222–288 (235)	185–199 (189)	155–181 (170)
BW	44–51 (45)	57–64 (59)	66–81 (73)	44–51 (46)	50–58 (53)
AOL	40–51 (49)	43–51 (47)	61–95 (68)	51–54 (51)	33–50 (45)
AOW	25–27 (27)	28–34 (31)	30–40 (34)	23–30 (27)	28–33 (30)
VSL	23–30 (29)	19–31 (28)	47–68 (53)	23–30 (25)	29–31 (31)
VSW	23–34 (28)	26–33 (30)	51–68 (59)	27–30 (28)	25–31 (27)
TSL	199–214 (206)	203–221 (213)	244–303 (273)	185–222 (211)	241–260 (250)
TW	29–31 (29)	21–32 (29)	40–44 (44)	37	30–34 (32)
FL	185–222 (201)	212–246 (232)	251–296 (273)	185–222 (208)	237–261 (252)
VSW/AOW	(1.0)	0.8–1.1 (1.0)	(1.7)	(1.0)	0.8–1.1 (0.9)
BL/TSL	(0.9)	0.7–0.8 (0.8)	(0.9)	(0.9)	0.6–0.7 (0.7)
TSL/FL	(1.0)	0.9–1.0 (0.9)	(1.0)	(1.0)	0.9–1.0 (1.0)

Data are presented as the range followed by the mean in parentheses. See Methods and Additional file 1: Figure S1 for description and illustration of the metrical features

Body armature: Pre-oral spines arranged in median group of 18 − 19 spines in 3 rows (Fig. 10c, Additional file 3: Figure S2G); spines in most anterior row largest, gradually decreasing in size in remaining rows; 2 lateral groups with 1 small spine each present. Post-oral spines more robust than spines on body, in 9 alternate rows; row 1 with median interruption; row 9 interrupted laterally; spines in row 1 larger than remaining spines, all of similar size. Wide zone of smaller, less dense, irregularly dispersed spines present posterior to post-oral spines, followed by narrow spineless area and 10 transverse rows of spines extending to mid-level of ventral sucker. Rows 1 − 8 complete (*i.e.* encircling body); rows 9 − 10 discontinuous ventrally; rows 1 − 2 doubled ventrally; row 3 with additional spines laterally. Two ventro-lateral fields of smaller spines (1.0–1.5) present posterior to ventral sucker; fields reach up to margin of ventral sucker and transverse row 10 laterally and dorsally and converge posterior to ventral sucker and close to posterior extremity of body. Ventral sucker armed with 3 rows of irregularly positioned spines (range 103 − 119; mean 110) (Fig. 10d, Additional file 4: Figure S3G). Tail stem and furcae with scale-like spines; spines on tail stem in 4 medio-lateral bands (2 ventral and 2 dorsal), each consisting of 3 scale-like spines (<1.0) (Fig. 10e); bands continue along furcal margins as rows of 4 spines anteriorly and single rows of spines posteriorly (Fig. 10f, Additional file 5: Figure S4G).

Resting position: Tail stem bent at < 45° (39°).

#### *Diplostomum pseudospathaceum* Niewiadomska, 1984

***First intermediate hosts***: *Lymnaea stagnalis* (Linnaeus); *Stagnicola palustris* (Müller).

***Localities***: Baldeneysee, Hengsteysee, Germany.

**Fig. 11:**
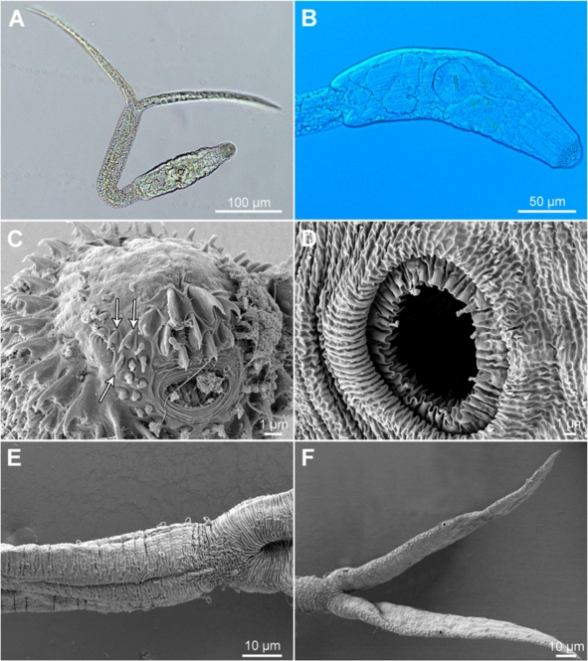
Cercaria of *Diplostomum pseudospathaceum* ex *Lymnaea stagnalis* (light and scanning electron microscopy, SEM). **a**, Resting position; **b**, Body; **c**, Anterior organ, apical view, arrows indicate group of lateral pre-oral spines (SEM); **d**, Ventral sucker (SEM); **e**, Tail stem and furcae (SEM); **f**, Furcae (SEM)

[Figure 11 and Additional file 3: Figure S2E, F, Additional file 4: Figure S3F, Additional file 5: Figure S4F. Measurements of formalin-fixed specimens are provided in Table 6.] Body elongate-oval, 152 − 183 × 46 − 54 (166 × 50), slightly shorter than tail stem [BL/TSL = 0.8 − 1.0 (0.9)] (Fig. 11a, b), with aggregations of yellow pigment in the parenchyma of whole body, concentrated on both sides of anterior organ, above ventral sucker and in tail stem and furcae. Anterior organ elongate-oval, with posterior margin reaching to mid-length of forebody, 41 − 58 × 22 − 28 (50 × 25). Ventral sucker spherical, small, somewhat post-equatorial, 24 − 32 × 27 − 32 (29 × 30), with fine undulating membrane (2 − 3 high) (Fig. 11d); width exceeds width of anterior organ [VSW/AOW = 1.1 − 1.4 (1.2)]. Penetration gland-cells 2 pairs, large, with fine granular content, posterior to ventral sucker, overlap caeca partially, posterior pair not reaching extremities of caeca. Tail stem 176 − 203 (187) long, 27 − 30 (29) wide at base, shorter than furcae [TSL/FL = 0.8 − 0.8 (0.8)], contains 35 − 45 caudal bodies; individual caudal bodies irregularly shaped with smooth contours. Furcae 219 − 253 (234) long, without fin-fold.

Body armature: Pre-oral spines arranged in median group of 10 − 11 spines in 3 rows; spines in anterior row largest, remaining spines of similar size; 2 lateral groups with 3 small spines each present (Fig. 11c, Additional file 3: Figure S2E, F). Post-oral spines more robust than spines on body, in 9 alternate rows; rows 1 − 2 with median interruption; row 9 interrupted laterally; first 2 spines in row 1 on both sides of median interruption largest; spines in row 1 larger than remaining spines, all of similar size. Wide zone of smaller, less dense, irregularly dispersed spines present posterior to post-oral spines, followed by narrow spineless area and 11 transverse rows of spines extending to mid-level of ventral sucker. Rows 1 − 8 complete (*i.e.* encircling body); row 9 discontinuous ventrally, rows 10 − 11 discontinuous ventrally and dorsally; rows 1 − 2 doubled ventrally; rows 3 − 7 with additional spines laterally. Two ventro-lateral fields of smaller spines (1.0–1.5) present posterior to ventral sucker; fields reach up to margin of ventral sucker and transverse row 11 laterally and dorsally and converge posterior of ventral sucker and close to posterior extremity of body. Ventral sucker armed with 2 rows of spines (*c.*42 per row; range 70 − 100; mean 84); third row may be partially formed (Fig. 11d, Additional file 4: Figure S3F). Tail stem and furcae with scale-like spines (Fig. 11e); spines on tail stem in 4 medio-lateral bands (2 ventral and 2 dorsal), each consisting of 1 − 2 scale-like spines anteriorly, 2 − 3 spines posteriorly, increasing in size posteriorly (0.5 − 1.3 μm); bands continue along furcal margins as rows of 3 spines anteriorly and 2 spines posteriorly (Fig. 11f, Additional file 5: Figure S4F).

Resting position: Tail stem bent at < 45° (29 − 38°).

## Discussion

To the best of our knowledge, this study provides the first combined morphological and molecular characterisation of *Diplostomum* spp. in natural lymnaeid snail populations in central Europe and is the first to apply thorough SEM analysis of species-specific features of the cercariae that can be used for species identification and delineation. This integrative approach allowed us to (i) provide evidence for morphological and molecular differentiation within the ‘*D. mergi*’ species complex, including a previously undetected lineage; (ii) clarify that ‘*D. mergi* Lineage 1’ of Georgieva *et al.* [6] represents *D. parviventosum*; (iii) partially elucidate the life-cycle of ‘*D. mergi* Lineage 3’ and ‘*Diplostomum* sp. Clade Q’ of Georgieva *et al.* [6]; (iv) expand the *cox*1 database for the European species *D. pseudospathaceum* and *D. spathaceum* in association with descriptions based on sequenced isolates; and (vii) assess the first intermediate host-specificity of *D. pseudospathaceum*.

Morphologically, the cercaria corresponding genetically to ‘*D. mergi* Lineage 1’ of Georgieva *et al.* [6] keyed down to *D. parviventosum* in the key of Niewiadomska & Kiselienė [10] due to the presence of 6–7 pre-oral spines, 11 transverse rows of spines on the body, 10–12 pairs of caudal bodies in the tail, ventral sucker slightly exceeding the width of the anterior organ [VSW/AOW 1.0–1.4 (1.2)], fin-folds on the furcae, and small penetration gland-cells which do not cover ends of caeca (see Additional file 6: Table S2). As described for *D. parviventosum* by Niewiadomska & Kiselienė [10], the present cercaria also shows a characteristic resting position with the tail stem bent at about 45° but differs in having 11 transverse rows of spines on the body (*vs* 10); of these rows 9–11 are interrupted ventrally and dorsally and rows 5–8 are interrupted dorsally (*vs* rows 6–10 with ventral and dorsal interruption) (Additional file 6: Table S2). Comparisons of the metrical data for fixed cercariae revealed differences probably due to the fixation method (Table 5). The present cercariae (both live and formalin-fixed) exhibit lower ranges and means for the length of the body, anterior organ and tail stem, the latter resulting in a lower range for the ratio TSL/FL (mean 0.8 *vs* 1.1), and higher upper limits for the width of the ventral sucker (46 *vs* 37 μm). However, the ratios VSW/AOW and BL/TSL are similar (see means in Table 5). Furthermore, the number of rows of post-oral spines (7–8 alternate rows) and the number of spines on the ventral sucker (77–87 in two rows) agree well with the description by Niewiadomska & Kiselienė [10], *i.e.* 6–8 rows of post-oral spines and 80–88 spines on ventral sucker in two rows (Additional file 6: Table S2). Our study thus provides the first detailed morphological description of the cercaria of *D. parviventosum* based on both light and scanning electron microscopy. Furthermore, the concordance of the morphological and molecular data clearly suggests that the isolates of ‘*D. mergi* Lineage 1’ of Georgieva *et al.* [6] actually belong to *D. parviventosum*. However, the ITS sequences for this species generated by us formed a reciprocally monophyletic lineage within the ‘*D. mergi*’ species complex instead of joining the cluster of sequences (‘Clade Q’ *sensu* Georgieva *et al.* [6]) representative for *D. parviventosum* according to Niewiadomska & Laskowski [23] (see also comment below).

Cercariae of the remaining three lineages of the ‘*D. mergi*’ species complex discovered in this study differ from the description of *D. mergi* by Niewiadomska & Kiselienė [10] in four unique qualitative features, *i.e.* the presence of scale-like spines on the tail stem and furcae and of yellow pigment in the forebody, the greater number of rows of post-oral spines (10–11 *vs* 6–9) and in the different number of transverse rows with additional spines laterally (Additional file 6: Table S2). Cercariae of Lineages 2 and 3 of ‘*D. mergi*’ can be further differentiated from *D. mergi* of Niewiadomska & Kiselienė [10]) by having furcae longer than tail stem (*vs* equal); the cercaria of ‘*D. mergi* Lineage 3’ also possesses 11 transverse rows of spines on body (*vs* 10) and a smaller number of spines on ventral sucker (90–92 *vs* 94–130) and the cercaria of *D. mergi* Lineage 4 is unique in having three pairs of penetration gland-cells (Additional file 6: Table S2). Morphometric comparisons revealed that the cercariae of *D. mergi* Lineage 4 exhibit much higher upper ranges and means for all morphometric characters than those of the two other lineages (Lineage 1 and 2; both live and fixed samples) and those in the description of *D. mergi* by Niewiadomska & Kiselienė [10] (Table 5). Fixed cercariae of Lineages 2 and 3 both differ from those of *D. mergi* as described by Niewiadomska & Kiselienė [10] in having generally shorter (mean length 48–49 *vs* 58 μm) and wider (mean width 32–33 *vs* 28 μm) anterior organs, narrower tail stems (mean 29–33 *vs* 36 μm), longer furcae (mean 216–225 *vs* 210 μm) that are also longer than tails (mean TSL/FL 0.8–0.9 *vs* 1.0), and shorter (‘*D. mergi* Lineage 2’)/longer (‘*D. mergi* Lineage 3’) tail stems (means 160 and 212 *vs* 207 μm, respectively). Further, compared with the cercaria of *D. mergi* described by Niewiadomska & Kiselienė [10], the cercaria of ‘*D. mergi* Lineage 2’ has a shorter body (mean 168 *vs* 182 μm), a wider ventral sucker (mean 53 *vs* 44 μm) and a greater ratio BL/TSL (mean 1.0 *vs* 0.9), and the cercaria of ‘*D. mergi* Lineage 3’ has a narrower body (mean 49 *vs* 59 μm) and a smaller ventral sucker (mean 31 × 32 *vs* 44 × 44 μm) that is also narrower in relation to the anterior organ (mean VSW/AOW 1.0 *vs* 1.6).

Overall, the isolates of the three lineages (Lineages 2–4) of the ‘*D. mergi*’ species complex described here exhibit a number of unique differentiating features (five for Lineages 2 and 4 and nine for Lineage 3; see Additional file 6: Table S2). In addition to the consistent differences in the morphometric characters and ratios (Table 5) the live cercariae of the single isolate of *D. mergi* Lineage 4 differ from those of both Lineage 2 and 3 in the relation BL < TSL = FL (*vs* BL ≤ TSL < FL and BL ≤ TSL < FL, respectively), in the pattern of incomplete transverse rows of spines on the body (ventral interruption in rows 8–10 *vs* 6–10 and 4–11, respectively; dorsal interruption in rows 5–10 *vs* 6–10 and 4–11, respectively), and in the lack of transverse rows with additional lateral spines.

Both live and fixed cercariae of ‘*D. mergi* Lineage 2′ differ from those of ‘*D. mergi* Lineage 3’ in having wider bodies (mean 63 and 64 *vs* 53 and 49 μm, respectively), larger ventral suckers (50 × 47 *vs* 31 × 31 μm and 46 × 53 *vs* 31 × 32 μm, respectively) that are also distinctly wider than anterior organs [ratio VSW/OSW 1.5–2.0 (1.7) *vs* 1.0–1.2 (1.1) and 1.5–2.1 (1.6) *vs* 0.9–1.1 (1.0), respectively]. The cercaria of ‘*D. mergi* Lineage 2’ further differs from the cercaria of ‘*D. mergi* Lineage 3’ in having 5–6 pre-oral spines of the median group located in 2 rows (*vs* 7 in 3 rows) with one spine very large, 11 rows of post-oral spines (*vs* 10) with spines in the first row larger than the remaining (*vs* spines in the first two rows), 10 transverse rows of spines (*vs* 11), as well as in the lack of double transverse rows (*vs* row 1), in the pattern of incomplete transverse rows and in having distinctly more spines on the ventral sucker (110–120 *vs* 90–92) (Additional file 6: Table S2). All these differences, in association with the molecular evidence, justify the distinct status of the four lineages of the ‘*D. mergi*’ species complex examined by us. However, it is difficult to decide whether the description of Niewiadomska & Kiselienė [10] (see Additional file 6: Table S2) corresponds to one of these due to the different level of detail provided in the early description of *D. mergi* and the consistent differences outlined above.

The detailed morphological and molecular data provided here further advance our knowledge of the ‘*D. mergi*’ species complex in several aspects. First, we have clarified that ‘*D. mergi* Lineage 1’ of Georgieva *et al.* [6] in fact represents *D. parviventosum*. These authors provided sequence data for a single isolate ex *R. auricularia* from Hengsteysee thus making decisions of its relationships difficult, whereas our study provides ample evidence for the distinct status of this lineage, its identification to the species level and the detection of its relatively high prevalence in *R. auricularia* in Hengsteysee, and probably elsewhere in Europe. Our study further expands the number of isolates of ‘*D. mergi* Lineage 2’ ex *R. auricularia* and its distribution in Baldeneysee, Hengsteysee and Sorpetalsperre. Finally, we provide the first link between sequences for ‘*D. mergi* Lineage 3’ from isolates of metacercariae in the second intermediate host (*Salmo trutta fario* and *Gobio gobio* from the River Ruhr; see Georgieva *et al.* [6]) and a number of isolates from the first intermediate hosts (*R. auricularia* from Hengsteysee) thus partially elucidating the life-cycle of this lineage (arguably species). Further efforts should be focused on the discovery of the adult stages and formal descriptions of the three novel lineages (Lineages 2, 3 and 4) of the ‘*D. mergi*’ species complex.

Two ITS1-5.8S-ITS2 sequences for *D. mergi* (*sensu lato*) have been published recently by Haarder *et al.* [18] from cercarial isolates ex *Radix balthica* (L.) in Denmark. These authors have shown experimentally that the cercariae infect *Oncorhynchus mykiss* (Walbaum). Faltýnková *et al.* [8] suggested, based on analysis of ITS1 only, that one of the isolates (JX494231) may belong to ‘*D. mergi* Lineage 2’ whereas the second (JX494233) appeared associated with ‘*D. mergi* Lineage 3’. In our analyses based on the entire ITS gene cluster one of the isolates (JX494231) clustered together with the single isolate of *D. mergi* Lineage 4 (however with low support) and the other clustered with isolates of ‘*D. mergi* Lineage 2’. Analysis of *cox*1 sequences for these two isolates would help reveal their actual assignment.

Our study expanded the *cox*1 database for European *D. pseudospathaceum* and *D. spathaceum* (18 and 7 isolates, respectively). The new isolates of both species clustered together with the isolates reported previously by Georgieva *et al.* [6] with high support. Based on all sequence data available to date, we can confidently suggest that *D. pseudospathaceum* completes its life-cycle using only *L. stagnalis* and *S. palustris* as first intermediate hosts and that the latter two hosts are infected only with this species. The two isolated records of *D. pseudospathaceum* ex *R. auricularia* (see [24, 25]) most probably represent misidentifications. The lack of infections with *D. pseudospathaceum* in more than 3,500 *R. auricularia* examined in the River Ruhr drainage ([26]; present study) provides further support for this suggestion. Morphologically, the cercarial isolates sequenced here generally (excluding the number of caudal bodies) key down to *D. pseudospathaceum* in the key of Niewiadomska & Kiselienė [10]. However, our detailed description (including SEM examination) of the cercaria revealed some differences compared with the data provided by these authors that generally show a wider range of variation: 10–11 pre-oral spines in the median group (*vs* 8–14); 3 pre-oral spines in each lateral group (*vs* 1–4); 9 post-oral rows of spines (*vs* 6–8); 11 transverse rows of spines on body (*vs* 10); transverse rows 3–7 with additional spines laterally (*vs* rows 3–4); spines present on entire tail stem (*vs* present at distal end of the tail stem); and resting position with tail stem bent at < 45° (*vs* at 90°) (see Additional file 6: Table S3). Morphometric comparisons revealed that both live and formalin-fixed cercariae described here possess shorter and wider bodies (means 166 × 50 and 162 × 59 μm, respectively, *vs* 185 × 45 μm), longer furcae (means 234 and 232 μm, respectively, *vs* 201 μm), the latter resulting in somewhat lower TSL/FL ratios (means 0.8 and 0.9, respectively, *vs* 1.0). Fixed cercariae described by us further exhibit greater width of the anterior organ (mean 31 *vs* 27 μm) and length of the tail stem (mean 213 *vs* 206 μm) and a lower BL/TSL ratio (mean 0.8 *vs* 0.9) (Table 6). These data indicate that SEM examination and adequate fixation should be considered for identification of the cercariae of *D. pseudospathaceum* in future studies.

This study is the first to provide a description of molecularly identified cercarial isolates of *D. spathaceum*. Both live and formalin-fixed isolates of *D. spathaceum* studied by us exhibit smaller dimensions for the size of the body, tail and all organs compared with the description of the cercaria of *D. spathaceum* by Niewiadomska [27] (the same data from 10 heat-fixed specimens were reiterated by Niewiadomska & Kiselienė [10]) (Table 6). Qualitative comparisons revealed that our isolates possess a slightly greater number of pre-oral spines (18–19 *vs* 8–16 in the median group and 1 in each lateral group *vs* no lateral spines), a smaller number of post-oral spines (9 *vs* 10–14), three spine rows on ventral sucker (*vs* 2), spined tail stem and furcae (*vs* unspined) and a smaller angle of bending of the tail stem in resting position (<45° *vs* 90°) (see Additional file 6: Table S3).

The cercaria of *D. spathaceum* described above exhibits similarities with the description of *D. paracaudum* by Niewiadomska [28] reiterated by Niewiadomska & Kiselienė [10] such as: an overlap in the number of the pre-oral spines (18–19 *vs* 15–20 spines in the median group; 1 *vs* 1–2 pre-oral spines in each lateral group) and the presence of 10 transverse rows of spines on the body, three (incomplete) spine rows on the ventral sucker and large penetration gland-cells that do not cover ends of the caeca (Additional file 6: Table S3). However, the present cercaria possesses yellow pigment in the body, a ventral sucker slightly wider than the anterior organ, nine (*vs* 6–7) rows of post-oral spines and a different pattern of spines in the transverse rows of spines on the body [rows 1–2 double ventrally only (*vs* row 1); rows 9–10 with ventral interruption (*vs* rows 5–10 with both ventral and dorsal interruption); row 3 with additional spines laterally (*vs* anteriormost rows)]. Further differences include the lower ranges of the number of spines on the ventral sucker (103–119 *vs* 116–141), the presence of spines on the tail stem and furcae (*vs* absent) and the much smaller angle of bending of the tail stem in resting position (<45° *vs* 90°) (Additional file 6: Table S3). Comparisons of the morphometric data revealed that both live and formalin-fixed isolates of *D. spathaceum* studied by us exhibit shorter and wider bodies (means 172 × 51 and 170 × 53 μm, respectively, *vs* 189 × 46 μm), longer ventral suckers (means 30 and 31 μm, respectively, *vs* 25 μm), longer (means 219 and 250 μm, respectively, *vs* 211 μm) but narrower tail stems (means 31 and 32 μm, respectively, *vs* 37 μm), much longer furcae (means 260 and 252 μm, respectively, *vs* 208 μm) and lower BL/TSL ratios (means 0.8 and 0.7, respectively, *vs* 0.9) (Table 6). The above comparisons indicate that the morphology of the cercaria of *D. spathaceum* characterised molecularly in the present study departs from the single limited descriptions of cercariae of both *D. spathaceum sensu* Niewiadomska [27] and *D. paracaudum sensu* Niewiadomska [28]. It is unfortunate that the morphologies described by Niewidomska have not been confirmed for nearly 30 years.

Georgieva *et al.* [6] denoted as ‘Clade Q’ (questionable) a single genotype representing two cercarial isolates ex *R. ovata* identified as *D. spathaceum* (AF419275; AF419276) and two for a cercarial isolate ex *R. ovata* identified as *D. parviventosum* (AF419277; AF419278) by Niewiadomska & Laskowski [23]; one metacercarial isolate ex *R. rutilus* submitted to GenBank as *D.* cf. *parviventosum*/*spathaceum* (JF775727) by Rellstab *et al.* [29]; and one cercarial isolate ex *R. auricularia* (JQ665458; isolate RA97) annotated as *D. mergi* in GenBank but published as *D. spathaceum* by Behrmann-Godel [17]. One additional metacercarial isolate belonging to ‘Clade Q’ has been recently sequenced and described by Pérez-del-Olmo *et al.* [9]. Georgieva *et al.* [6] also suggested to use temporarily the name *D. parviventosum* as a label for the four identical sequences (AF419275–AF419278) of Niewiadomska & Laskowski [23]. One of the important results of our integrative taxonomic approach is the clarification of the distinct status of *D. parviventosum* and its close relationship with the species/lineages of the ‘*D. mergi*’ species complex (see above). However, the species identification of the sequences within ‘Clade Q’ *sensu* Georgieva *et al.* [6] is still questionable. The fact that new isolates are being added to this clade ([9, 17]; present study) indicates that this lineage has a wider distribution and still requires taxonomic scrutiny. It is worth noting that the metacercaria of ‘*Diplostomum* sp. Clade Q’ ex *Cyprinus carpio* L. from the Ebro Delta in Spain described and sequenced by Pérez-del-Olmo *et al.* [9] was shown to possess a smaller oral sucker and a shorter holdfast organ compared with the Spanish and Polish (see Niewiadomska [27]) isolates of *D. spathaceum* plus a distinctly lower number of excretory granules in the secondary excretory system than the metacercariae of *D. spathaceum sensu* Niewiadomska [27]. The cercaria of the single isolate of ‘*Diplostomum* sp. Clade Q’ sequenced and described here keys down to *D. spathaceum* in the key by Niewiadomska & Kiselienė [10] and agrees with their description in many aspects. However, the present cercaria differs in having fewer pre-oral spines in the median group (9 *vs* 8–16), 12 post-oral rows of spines (*vs* 10–14), 10 pairs of caudal bodies (*vs* 11–12 pairs), as well as in the presence of two non-converging fields of dispersed spines in the hindbody (*vs* two fields converging ventrally) and of bands of scale-like spines on the tail stem and furcae (*vs* spines absent); spines on the latter enveloped by tegumental membrane forming a specific fish-fin like fin-fold (Additional file 6: Table S3). Although the metrical data for our isolate (live cercariae measured only) are not directly comparable with those by Niewiadomska [27] the former exhibits much lower values for the length of body (mean 224 *vs* 235 μm) and the ratios VSW/AOW (mean 1.3 *vs* 1.7) and BL/TSL (mean 0.8 *vs* 0.9) and much greater values for body width (mean 96 *vs* 73 μm) and for the size of the anterior organ (mean 80 × 47 *vs* 68 × 34 μm) and ventral sucker (mean 56 × 65 *vs* 53 × 59 μm). The above comparisons indicate that the cercariae and metacercariae of ‘*Diplostomum* sp. Clade Q’ possess distinctive morphological characteristics that do not allow their identification as *D. spathaceum sensu* Niewiadomska [27]. The solution for the taxonomic status of this clade should await morphological and molecular data for the adult stages.

## Conclusion

The integration of molecular and morphological evidence for *Diplostomum* spp. achieved in this study will serve as a baseline for species identification of these important parasites of snail and fish populations and thus advance further studies on the distribution of *Diplostomum* spp. in Europe.

## Additional files

Additional file 1: Figure S1. Schematic illustration of a cercaria of *Diplostomum* spp. showing the metrical features used. *Abbreviations*: BL, body length; BW, maximum body width; AOL, anterior organ length; AOW, anterior organ maximum width; VSL, ventral sucker length; VSW, ventral sucker width; TSL, tail stem length; TSW, tail stem width (at base); FL, furca length. Additional file 2: Table S1. Summary data for *cox*1 sequences for *Diplostomum* spp. retrieved from GenBank. Additional file 3: Figure S2. Cercariae of *Diplostomum* spp. Pre- and post-oral spines (light microscopy). A, *Diplostomum parviventosum*; B, *Diplostomum mergi* Lineage 4; C, ‘*Diplostomum mergi* Lineage 2’; D, ‘*Diplostomum mergi* Lineage 3’; E, F, *Diplostomum pseudospathaceum* (arrows indicate lateral spines); G. *Diplostomum spathaceum*. Additional file 4: Figure S3. Cercariae of *Diplostomum* spp. Ventral sucker (light microscopy). A, *Diplostomum parviventosum*; B, *Diplostomum mergi* Lineage 4; C, ‘*Diplostomum mergi* Lineage 2’; D, ‘*Diplostomum mergi* Lineage 3’; E, ‘*Diplostomum* sp. Clade Q’; F, *Diplostomum pseudospathaceum*; G. *Diplostomum spathaceum*. Additional file 5: Figure S4. Cercariae of *Diplostomum* spp. Tail furcae (light microscopy). A, *Diplostomum parviventosum*; B, *Diplostomum mergi* Lineage 4; C, ‘*Diplostomum mergi* Lineage 2’; D, ‘*Diplostomum mergi* Lineage 3’; E, ‘*Diplostomum* sp. Clade Q’; F, *Diplostomum pseudospathaceum*; G. *Diplostomum spathaceum*. Additional file 6: Table S2. Comparative qualitative and meristic data for cercariae of the *Diplostomum ‘mergi’* species complex. **Table S3** Comparative qualitative and meristic data for cercariae of *Diplostomum spathaceum*, *D. pseudospathaceum*, *D. paracaudum* and ‘*Diplostomum* sp. Clade Q’ of Georgieva *et al.* [6].
