# Supplementary material for: Integrative taxonomic approach to the cryptic diversity of Diplostomum spp. in lymnaeid snails from Europe with a focus on the ‘Diplostomum mergi’ species complex
Source: Parasit Vectors. 2015 Jun 3;8:300. doi: 10.1186/s13071-015-0904-4 (PMC4476078; doi:10.1186/s13071-015-0904-4)
Supplement: Additional file 1: Figure S1. — Schematic illustration of a cercaria of Diplostomum spp. showing the metrical features used. Abbreviations: BL, body length; BW, maximum body width; AOL, anterior organ length; AOW, anterior organ maximum width; VSL, ventral sucker length; VSW, ventral sucker width; TSL, tail stem length; TSW, tail stem width (at base); FL, furca length. [file 13071_2015_904_MOESM1_ESM.pdf]

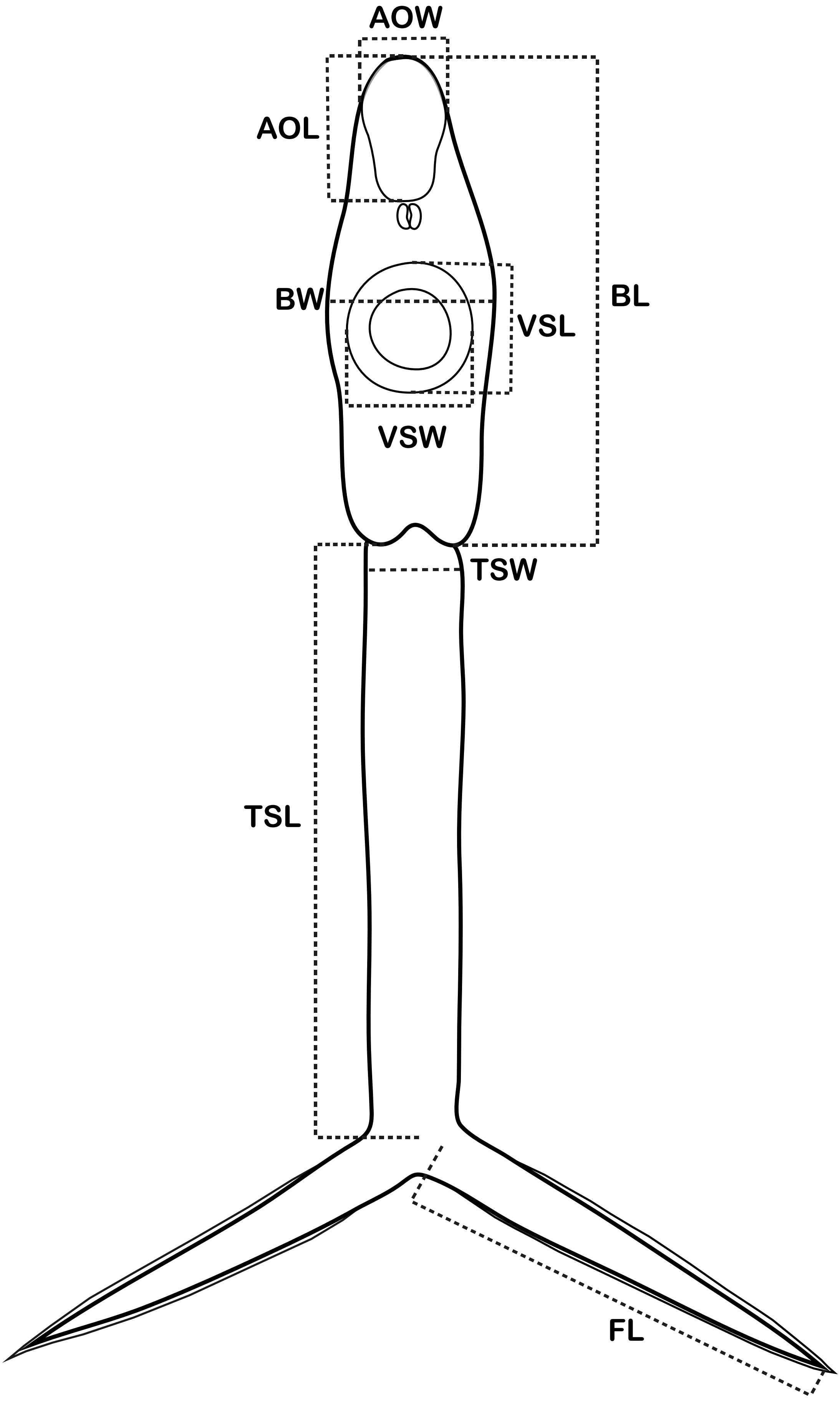

Supplementary Figure S1 Schematic illustration of a cercaria of *Diplostomum* spp. showing the metrical features used. *Abbreviations*: BL, body length; BW, maximum body width; AOL, anterior organ length; AOW, anterior organ maximum width; VSL, ventral sucker length; VSW, ventral sucker width; TSL, tail stem length; TSW, tail stem width (at base); FL, furca length.
