# Supplementary material for: Integrative taxonomic approach to the cryptic diversity of Diplostomum spp. in lymnaeid snails from Europe with a focus on the ‘Diplostomum mergi’ species complex
Source: Parasit Vectors. 2015 Jun 3;8:300. doi: 10.1186/s13071-015-0904-4 (PMC4476078; doi:10.1186/s13071-015-0904-4)
Supplement: Additional file 4: Figure S3. — Cercariae of Diplostomum spp. Ventral sucker (light microscopy). A, Diplostomum parviventosum; B, Diplostomum mergi Lineage 4; C, ‘Diplostomum mergi Lineage 2’; D, ‘Diplostomum mergi Lineage 3’; E, ‘Diplostomum sp. Clade Q’; F, Diplostomum pseudospathaceum; G. Diplostomum spathaceum. [file 13071_2015_904_MOESM4_ESM.pdf]

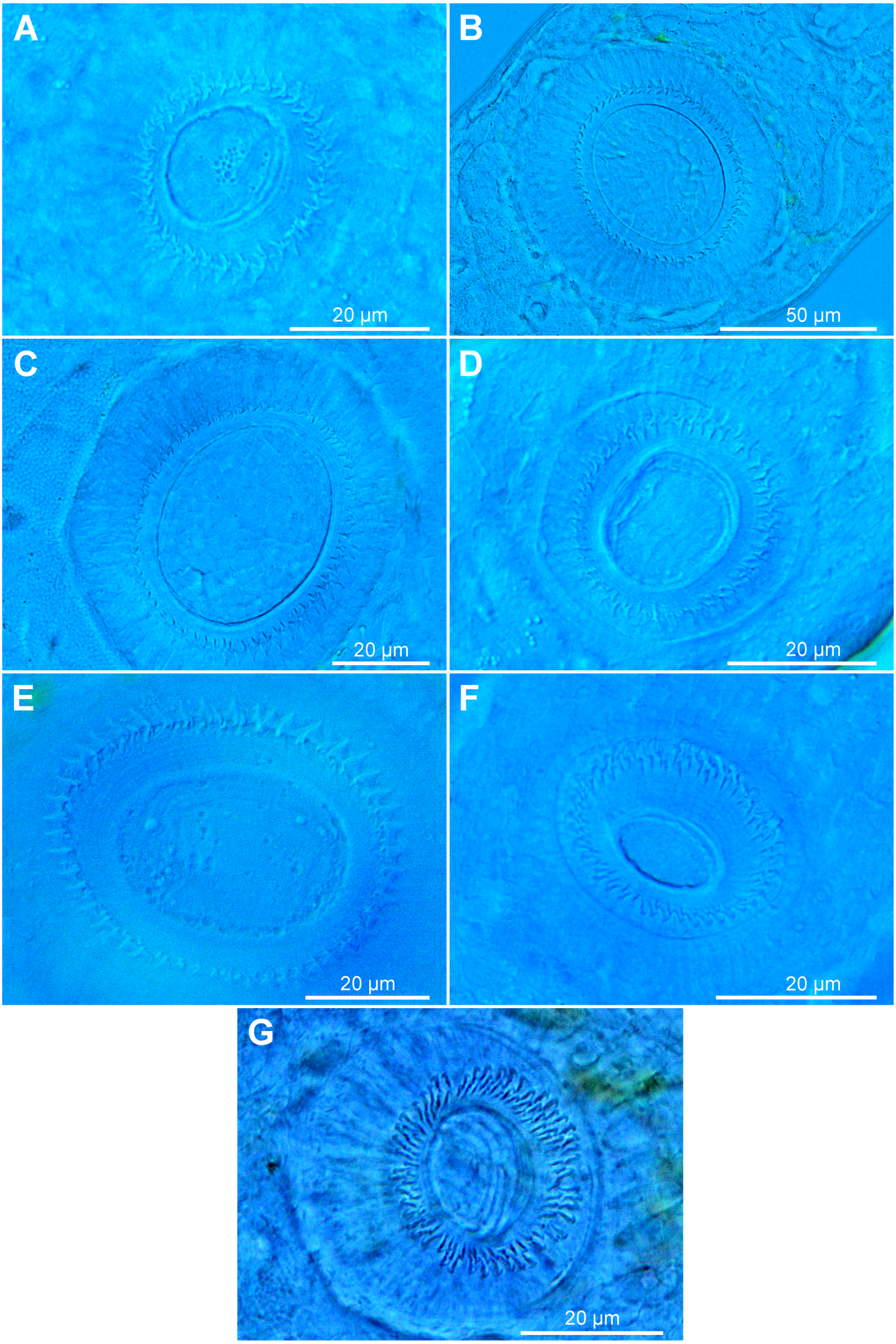

Supplementary Figure S3 Cercariae of *Diplostomum* spp. Ventral sucker (light microscopy). A, *Diplostomum parviventosum*; B, *Diplostomum mergi* Lineage 4; C, '*Diplostomum mergi* Lineage 2'; D, '*Diplostomum mergi* Lineage 3'; E, '*Diplostomum* sp. Clade Q'; F, *Diplostomum pseudospathaceum*; G. *Diplostomum spathaceum*
