# Supplementary material for: Integrative taxonomic approach to the cryptic diversity of Diplostomum spp. in lymnaeid snails from Europe with a focus on the ‘Diplostomum mergi’ species complex
Source: Parasit Vectors. 2015 Jun 3;8:300. doi: 10.1186/s13071-015-0904-4 (PMC4476078; doi:10.1186/s13071-015-0904-4)
Supplement: Additional file 6: Table S2. — Comparative qualitative and meristic data for cercariae of the Diplostomum ‘mergi’ species complex. Table S3 Comparative qualitative and meristic data for cercariae of Diplostomum spathaceum, D. pseudospathaceum, D. paracaudum and ‘Diplostomum sp. Clade Q’ of Georgieva et al. [6]. [file 13071_2015_904_MOESM6_ESM.pdf]

Electronic supplementary material for

Integrative taxonomic approach to the cryptic diversity of *Diplostomum* spp. in lymnaeid snails from Europe with a focus on the '*Diplostomum mergi*' species complex

Christian Selbach[1*], Miroslava Soldánová[2], Simona Georgieva[2], Aneta Kostadinova[2] and Bernd Sures[1, 3]

[1]Department of Aquatic Ecology and Centre for Water and Environmental Research (ZWU), University of Duisburg-Essen, Universitätsstraße 5, Essen D-45141, Germany

[2]Institute of Parasitology, Biology Centre of the Czech Academy of Sciences, Branišovská 31, 370 05 České Budějovice, Czech Republic

[3]Department of Zoology, University of Johannesburg, PO Box 524, Auckland Park 2006, Johannesburg, South Africa

***Corresponding author. E-mail address: christian.selbach@uni-due.de**

Table S2 Comparative qualitative and meristic data for cercariae of the *Diplostomum* ‘*mergi*’ species complex

Species	*Diplostomum parviventosum* Dubois, 1932		*Diplostomum mergi* Dubois, 1932	‘*Diplostomum mergi* Lineage 2’ of Georgieva *et al.* [6]	‘*Diplostomum mergi* Lineage 3’ of Georgieva *et al.* [6]	*Diplostomum mergi* Lineage 4
Hosts	*Radix auricularia*	*Radix ovata*	*R. ovata*	*R. auricularia*	*R. auricularia*	*R. auricularia*
Source	Present study	Niewiadomska & Kiseliene [10]	Niewiadomska & Kiseliene [10]	Present study	Present study	Present study
Yellow pigment in body	Present	na	Absent	Present	Present	Present
Relation BL-TSL-FL	BL<TSL<FL	BL<TSL>FL	BL<TSL=FL	Live: BL≤TSL<FL<br>Fixed: BL=TSL<FL	Live: BL≤TSL<FL<br>Fixed: BL<TSL≤FL	Live: BL<TSL=FL
Relation VSW-AOW	VSW>AOW	VSW>AOW	VSW>AOW	VSW>AOW	Live: VSW≥AOW<br>Fixed: VSW=AOW	Live: VSW>AOW
No. of pre-oral spines (median group)	6–7 in 3 rows	5–7 in 2 rows	6–8 in 2 rows	5–6 in 2 rows	7 in 3 rows	7 in 3 rows
No. of pre-oral spines in each lateral group	No lateral group	No lateral group	No lateral group	No lateral group	No lateral group	No lateral group
No. of rows of post-oral spines	7–8	6–8	6–9	11 (an additional median row may be present)	10	11
Incomplete rows of post-oral spines	Rows 1–2 with median interruption	na	na	Rows 1–2 with median interruption	Rows 1–2 with median interruption	Rows 1–2 with median interruption, rows 10–11 interrupted dorsally
Size of post-oral spines	First 4 spines in row 1 on both sides of median interruption largest; spines in row 1 larger than remaining spines	na	na	First 4 spines in row 1 and first 3 spines in row 2 on both sides of median interruption largest; spines in row 1 larger than remaining spines	First 4 spines in row 1 on both sides of median interruption largest; spines in rows 1–2 larger than remaining spines	First 4 spines in row 1 and first 3 spines in row 2 on both sides of median interruption largest; spines in rows 1–2 larger than remaining spines
Zone of dispersed post-oral spines	Present (wide)	Present	Present	Present (wide)	Present (wide)	Present (wide)
Spineless area posterior to dispersed spines	Present (narrow)	Present	Present	Present (narrow)	Present (narrow)	Present (narrow)
Transverse rows of spines on body	11	10	10	10	11	10
Double transverse rows	None	na	na	None	Row 1	None
Incomplete transverse rows	Rows 9–11 discontinuous ventrally and dorsally; rows 5–8 discontinuous dorsally	Rows 6–10 discontinuous ventrally and dorsally	Last rows	Rows 6–10 discontinuous ventrally and dorsally	Rows 4–11 discontinuous ventrally and dorsally	Rows 5–7 discontinuous dorsally; rows 8–10 discontinuous ventrally and dorsally
Transverse rows with additional spines laterally	Rows 1 and 10	na	Rows 1–2	Rows 1–4 (rows 5–6 with 1–2 additional spines)	Rows 2–3	None

Species	*Diplostomum parviventosum* Dubois, 1932		*Diplostomum mergi* Dubois, 1932	'*Diplostomum mergi* Lineage 2' of Georgieva *et al.* [6]	'*Diplostomum mergi* Lineage 3' of Georgieva *et al.* [6]	*Diplostomum mergi* Lineage 4
Hosts	*Radix auricularia*	*Radix ovata*	*R. ovata*	*R. auricularia*	*R. auricularia*	*R. auricularia*
Source	Present study	Niewiadomska & Kiseliene [10]	Niewiadomska & Kiseliene [10]	Present study	Present study	Present study
Zone of dispersed spines in hind body	2 fields converging posteriorly	2 wide ventro-lateral bands converging posterior to VS and close to posterior extremity	na	2 fields converging posteriorly	2 wide, non-converging fields	2 fields converging posteriorly
No. of spine rows on ventral sucker	2	2	2	2	2	2
No. of spines on ventral sucker (mean)	77–87 (81)	80–88	94–130	110–120 (114)	90–92 (90)	112–114 (113)
Penetration gland-cells	2 pairs; small, do not cover ends of caeca	2 pairs; small, do not overpass caeca laterally	2 pairs; large, do not cover ends of caeca	2 pairs; large, do not cover ends of caeca	2 pairs; medium-sized, do not cover ends of caeca	3 pairs (anterior pair small, posterior 2 pairs large); do not cover ends of caeca
Spines on tail stem	Present (2 ventral and 2 dorsal bands)	Dispersed spines on ventral and dorsal surfaces	Absent	Present (2 ventral and 2 dorsal bands) start from second quarter of tail	Present (2 ventral and 2 dorsal bands)	Present (2 ventral and 2 dorsal bands)
Spines on furcae	Present	Present	Absent	Present	Present	Present
Fin-folds on furcae	Present (fish-fin like fin-folds)	Present	Present	Present (fish-fin like fin-folds)	Present (fish-fin like fin-folds)	Present (fishfin-like fin-fold)
No. of caudal bodies	10–12 pairs	10–11 pairs	10–11 pairs	36–40 individual caudal bodies	Individual caudal bodies impossible to count	36–40 individual caudal bodies
Shape of caudal bodies	With smooth contours	With incised contours	With incised contours	With smooth contours	With incised contours	With smooth contours
Resting position	tail stem bent at < 90° (45–67°)	Tail stem bent at *c.*45°	Tail stem bent at < 90°	Tail stem bent at < 90° (64–85°)	Tail stem bent at 90° (77–91°)	Tail stem bent at < 90° (66°)

Abbreviations: BL, body length; BW, maximum body width; AOW, anterior organ width; VS, ventral sucker; VSW, ventral sucker width; TSL, tail stem length; FL, furca length; na, no data available

Table S3 Comparative qualitative and meristic data for cercariae of *Diplostomum spathaceum*, *D. pseudospathaceum*, *D. paracaudum* and '*Diplostomum* sp. Clade Q' of Georgieva *et al.* [6]

Species	*D. spathaceum* (Rudolphi, 1819)	*D. paracaudum* (Iles, 1959)	'*Diplostomum* sp. Clade Q' of Georgieva *et al.* [6]	*Diplostomum spathaceum* (Rudolphi, 1819)	*Diplostomum pseudospathaceum* Niewiadomska, 1984	
Hosts	*R. auricularia*, *R. ovata*, *R. peregra*	*R. auricularia*, *R. ovata*, *S. palustris*	*R. auricularia*	*R. auricularia*	*L. stagnalis*, *S. palustris*	*L. stagnalis*, *S. palustris*
Source	Niewiadomska & Kiseliene [10]	Niewiadomska [28]; Niewiadomska & Kiseliene [10]	Present study	Present study	Niewiadomska & Kiseliene [10]	Present study
Yellow pigment in body	Present	Absent	Present	Present	Present	Present
Relation BL-TSL-FL	BL<TSL=FL	BL<TSL≥FL	Live: BL<TSL=FL	Live: BL<TSL<FL<br>Fixed: BL<TSL=FL	BL<TSL=FL	Live: BL≤TSL<FL<br>Fixed: BL<TSL≤FL
Relation VSW-AOW	VSW>AOW	VSW=AOW	Live: VSW>AOW	Live: VSW≥AOW<br>Fixed: VSW≤AOW	VSW=AOW	Live: VSW>AOW<br>Fixed: VSW=AOW
No. of pre-oral spines (median group)	8–16 in 3–4 rows	15–20 in 3 rows	9 in 3 rows	18–19 in 3 rows	8–14 in a triangle	10–11 in 3 rows
No. of pre-oral spines in each lateral group	No lateral groups	1–2	No lateral groups	1 small spine	1–4	3 small spines
No. of rows of post-oral spines	10–14	6–7	12	9	6–8	9
Incomplete rows of post-oral spines	na	na	Row 1 with median interruption, rows 11–12 interrupted laterally	Row 1 with median interruption; row 9 interrupted laterally	na	Rows 1–2 with median interruption; row 9 interrupted laterally
Size of post-oral spines	na	na	First 5 spines in row 1 on both sides of median interruption largest; remaining spines of different sizes	Spines in row 1 larger than remaining spines	na	Spines in row 1 larger than remaining spines
Zone of dispersed post-oral spines	Present	Present	Present (wide)	Present (wide)	Present	Present (wide)
Spineless area posterior to dispersed spines	Present	Present	Present (narrow)	Present (narrow)	Present	Present (narrow)
Transverse rows of spines on body	9–10	10	10	10	10	11
Double transverse rows	Rows 1–2	Row 1 (drawing)	Row 1	Rows 1–2 (only ventrally)	Rows 1–2	Rows 1–2 (only ventrally)
Incomplete transverse rows	Posterior rows discontinuous ventrally and dorsally	Rows 5–6 to 10 discontinuous ventrally and dorsally	Rows 5–10 discontinuous ventrally and dorsally	Rows 9–10 discontinuous ventrally	Last rows	Row 9 discontinuous ventrally; rows 10–11 discontinuous ventrally and dorsally

Species	*D. spathaceum* (Rudolphi, 1819)	*D. paracaudum* (Iles, 1959)	'*Diplostomum* sp. Clade Q' of Georgieva *et al.* [6]	*Diplostomum spathaceum* (Rudolphi, 1819)	*Diplostomum pseudospathaceum* Niewiadomska, 1984	
Hosts	*R. auricularia*, *R. ovata*, *R. peregra*	*R. auricularia*, *R. ovata*, *S. palustris*	*R. auricularia*	*R. auricularia*	*L. stagnalis*, *S. palustris*	*L. stagnalis*, *S. palustris*
Source	Niewiadomska & Kiseliene [10]	Niewiadomska [28]; Niewiadomska & Kiseliene [10]	Present study	Present study	Niewiadomska & Kiseliene [10]	Present study
Transverse rows with additional spines laterally	Rows 3–4	Anteriormost rows	Rows 2–3	Row 3	Rows 3–4	Rows 3–7
Zone of dispersed spines in hind body	2 fields converging ventrally	2 fields converging ventrally and dorsally	2 non-converging fields posterior to VS	2 fields converging posteriorly to VS and close to posterior extremity of body	2 lateral fields	2 fields converging posteriorly to VS and close to posterior extremity of body
No. of spine rows on ventral sucker	2	3	2	3	2 (3rd row may be partly formed)	2
No. of spines on ventral sucker (mean)	108–125	116–141	112–116 (114)	103–119 (110)	66–107	70–100 (84)
Penetration gland-cells	Large, do not cover ends of caeca	Large, do not cover ends of caeca	Large, do not cover ends of caeca	Large, do not cover ends of caeca	Large, do not cover ends of caeca	Large, do not cover ends of caeca
Spines on tail stem	Absent	Absent	Present (2 ventral and 2 dorsal bands)	Present (2 ventral and 2 dorsal bands)	Present near distal end	Present (2 ventral and 2 dorsal bands)
Spines on furcae	Absent	Absent	Present	Present	Present	Present
Fin-folds on furcae	Present	Absent	Present (fish-fin like fin-folds)	Absent	Absent	Absent
No. of caudal bodies	11–12 pairs	10–11 pairs	10 pairs	56–60 individual caudal bodies	10 pairs	35–45 individual caudal bodies
Shape of caudal bodies	With incised contours	With incised contours	With incised contours	With both incised and smooth contours	With incised contours	With smooth contours, irregular in shape and size
Resting position	Tail stem bent at 90°	Tail stem bent at 90°	na	Tail stem bent at < 45° (39°)	Tail stem bent at 90°	Tail stem bent at < 45° (29–38°)

Abbreviations: BL, body length; BW, maximum body width; AOW, anterior organ width; VS, ventral sucker; VSW, ventral sucker width; TSL, tail stem length; FL, furca length; na, no data available
